# Small RNA-modulated anaerobic respiration allows bacteria to survive under antibiotic stress conditions

**DOI:** 10.3389/fcimb.2024.1287557

**Published:** 2024-03-13

**Authors:** Dajeong Kim, Abhayprasad Bhat, Seon-Kyu Kim, Soohyun Lee, Choong-Min Ryu

**Affiliations:** ^1^ Molecular Phytobacteriology Laboratory, Infectious Disease Research Center, Korea Research Institute of Bioscience and Biotechnology, Daejeon, Republic of Korea; ^2^ Department of Microbiology, Tumor and Cell Biology, Centre for Translational Microbiome Research, Karolinska Institutet, Stockholm, Sweden; ^3^ Personalised Genomic Medicine Research Center, Korea Research Institute of Bioscience and Biotechnology, Daejeon, Republic of Korea; ^4^ Department of Pediatrics School of Medicine, University of California at San Diego, La Jolla, CA, United States

**Keywords:** regulatory RNAs, small RNAs, antibiotics, RNA-seq, anaerobic adaptation, cellular homeostasis

## Abstract

Despite extensive knowledge of antibiotic-targeted bacterial cell death, deeper understanding of antibiotic tolerance mechanisms is necessary to combat multi-drug resistance in the global healthcare settings. Regulatory RNAs in bacteria control important cellular processes such as cell division, cellular respiration, metabolism, and virulence. Here, we investigated how exposing *Escherichia coli* to the moderately effective first-generation antibiotic cephalothin alters transcriptional and post-transcriptional dynamics. Bacteria switched from active aerobic respiration to anaerobic adaptation via an FnrS and Tp2 small RNA-mediated post-transcriptional regulatory circuit. From the early hours of antibiotic exposure, FnrS was involved in regulating reactive oxygen species levels, and delayed oxygen consumption in bacteria. We demonstrated that bacteria strive to maintain cellular homeostasis via sRNA-mediated sudden respiratory changes upon sublethal antibiotic exposure.

## Introduction

The extensive use and misuse of antibiotics in human activities has led to the widespread distribution of antibiotics in nature, which in turn has induced the selection of multi-drug-resistant bacteria in the environment along with the resurgence of old-world infectious diseases which are of serious global health concern (Mahoney et al., 2021; Andersson and Hughes, 2014). Moreover, the overuse of antibiotics during the Coronavirus disease (COVID-19) pandemic accelerated the emergence and spread of antimicrobial resistant bacteria (Rawson et al., 2020). Antibiotics are classified based on the nature of their molecular targets such as DNA replication, protein synthesis, and cell wall biosynthesis (Keren et al., 2013). Interestingly, increasing evidence suggests that regulatory RNAs modulate antibiotic resistance by controlling the mRNA levels of genes involved in antibiotic resistance (Dersch et al., 2017; Mediati et al., 2021). In *Escherichia coli*, the small RNA (sRNA) MicF represses translation of the OmpF porin, which is an important route for the uptake of antibiotics (Nikaido, 1989). The sRNA GcvB also represses CycA mRNA, which encodes the D-cycloserine transporter CycA (Pulvermacher et al., 2009). The sRNA MgrR in *E. coli* suppresses translation of EtpB, which adds phosphoethanolamine to the keto-deoxyoctulosonate core of LPS (Moon and Gottesman, 2009). When phosphoethanolamine is added, the negative charge of LPS is reduced, thereby reducing interaction with polymyxin B and allowing *E. coli* to develop resistance to the drug.

However, recent reports suggest that antibiotics induce perturbations in secondary targets that are linked unequivocally to bacterial cellular respiration and metabolic reprogramming (Dwyer et al., 2014; Melior et al., 2021). In particular, a recent study demonstrated that *Listeria monocytogenes* switches to anaerobic adaptation upon exposure to four types of antibiotic at sublethal concentrations (ampicillin, tetracycline, gentamicin, and co-trimoxazole), leading to emergence of highly drug-tolerant cells (Knudsen et al., 2016); however, the molecular determinants and mechanisms underlying such abrupt cellular reprogramming in bacteria remain elusive.

In *E. coli*, alterations in expression of genes linked to the shift from aerobic to anaerobic metabolism are influenced by two transcriptional regulators, ArcA (aerobic respiratory control) and FNR (fumarate and nitrate reduction), the activities of which are modulated by oxygen availability (Shalel Levanon et al., 2005). Both ArcA and FNR activate many genes encoding anaerobic pathway enzymes while at the same time repressing a number of genes with aerobic activity (Durand and Storz, 2010). ArcA and FNR also activate expression of the sRNA FnrS (FNR-regulated sRNA) in anaerobic environments. FnrS functions to downregulate at least 32 mRNAs to increase the efficiency of anaerobic metabolism (Durand and Storz, 2010). Among these, expression of MarA is repressed by sRNA FnrS. In *E. coli*, MarA is a global regulator that controls expression of genes involved in resistance to antibiotics, oxidative stress, heavy metals, and chemical solvents (Rodionova et al., 2022).

Here, we use a transcriptomic analysis approach to show that treatment with sublethal concentrations of antibiotics increases expression of genes linked to anaerobic respiration. Transition of bacterial cells to anaerobic respiration was dependent on both ArcA and FNR. We found that FnrS and Tp2 were controlled by FNR and ArcA. Moreover, FnrS was involved in generation of reactive oxygen species (ROS), as well as delayed oxygen consumption, by *E. coli*.

## Materials and methods

### Strains, media, and growth conditions

The isogenic *Escherichia coli (E. coli)* strains, plasmids, and primers used in this study are listed in **Supplementary Table S4**. The *E. coli Δfnr* and *ΔarcA* mutants were obtained from the Keio collection (Baba et al., 2006), and the *ΔfnrS* mutant and FnrS-overexpressing pBRplac plasmid systems were kind gifts from Gisela Storz (Durand and Storz, 2010) and Susan Gottesman (Guillier and Gottesman, 2006), respectively. *E. coli* cells were cultured in Luria Bertani (LB) or M9 minimal media supplemented with 0.2% casamino acids and 10 mM glucose. In all experiments, cells were grown at 37°C, either in rotating shakers or in a Whitley DG250 anaerobic workstation.

### Antibiotics and chemicals

*E. coli* cells were treated with 1/4× minimal inhibitory concentration (MIC) of Cephalothin (Ceph; 40 µg/mL). When necessary, 20 µg/mL of Ceph was added as indicated. For plasmid selection, ampicillin at 100 µg/mL and kanamycin at 50 µg/mL were used. All antibiotics were purchased from Sigma.

### MIC determination and growth inhibition test

MICs of the tested antibiotics were determined by microbroth dilution method (Im et al., 2016). In brief, overnight cultures of *E. coli* MG1655 were freshly inoculated into a 125-ml flask containing 20 ml of LB medium at a 1:100 dilution and allowed to grow until an OD_600_ of 0.3 in a 37°C incubator with shaking at 200 rpm. A total of 0.5% of the cultures were used to serially dilute antibiotics (2-fold) and incubated at 37°C in well-aerated conditions (200 rpm). OD_600_ values were measured in triplicate from between 0 and 24 h using a Tecan Infinite 200 Pro multimode reader (Tecan Austria GmbH, Grodig, Austria).

### Total RNA extraction

Overnight cultures of *E. coli* MG1655 containing the pBAD-*ryhB* plasmid were diluted to an OD_600_ of 0.05 using LB media and allowed to grow until the OD_600_ reached 0.5. All cultures were prepared without induction. Cells were then treated with 1/4× Ceph (40 µg/ml), 10 ml of the culture was harvested at OD_600_ 0.6, and an equal volume of hot phenol was immediately added and thoroughly mixed. Total RNA extraction was performed using the hot phenol method (Aiba et al., 1981). RNA-sequencing (RNA-seq) was performed using an aliquot of prepared total RNA. Another aliquot was used for and sRNA-sequencing (sRNA-seq). Wild-type MG1655 or BW25113 were used for qRT-polymerase chain reaction (PCR), biomass and dissolved oxygen measurement, and the ROS detection assay.

### RNA-seq

Libraries were prepared for 100 bp paired-end sequencing using a TruSeq RNA Sample Preparation Kit (Illumina, CA, USA). Briefly, mRNA molecules were fragmented from 2 μg total RNA using divalent cations under elevated temperature. Fragmented mRNAs were converted into single-stranded cDNAs through random hexamer priming. By applying products as a template for second strand synthesis, double-stranded cDNA was prepared. To produce the final cDNA library, the products were purified and enriched using PCR. The quality of cDNA libraries was evaluated with an Agilent 2100 BioAnalyzer (Agilent, CA, USA), and quantification was performed with a KAPA library quantification kit (Kapa Biosystems, MA, USA) according to the manufacturer’s instructions. Following cluster amplification of denatured templates, paired-end (2×100 bp) sequencing was performed using an Illumina HiSeq2500 instrument (Illumina, CA, USA).

### Transcriptome data analysis

#### Filtering

Low-quality reads were filtered according to the following criteria: reads containing more than 10% of skipped bases (marked as ‘N’s), reads containing more than 40% of bases whose quality scores were less than 20 and reads for which the average quality score of each read was less than 20. The whole filtering process was performed using in-house scripts.

#### Sequence alignment

Filtered reads were mapped to the reference genome of the appropriate species using the aligner TopHat (Trapnell et al., 2009).

#### Gene expression estimation

Gene expression levels were measured with Cufflinks v2.1.1 (Trapnell et al., 2010) using the gene annotation database of the relevant species. The non-coding gene region was excluded from gene expression measurements using the mask option. To improve the accuracy of the measurement, multi-read- correction and frag-bias-correct options were applied. All other options were set to default values.

#### Differentially expressed gene analysis

Using an Illumina HiSeq 2500 platform, more than 50 million clean reads were obtained from two generated cDNA libraries. Of these, 89.6% (49,639,165) of genes in the untreated sample and 88.8% (49,457,597) of genes in the cephalothin-treated sample were uniquely mapped using TopHat aligner, with a reliable sample coverage of 96% (**Supplementary Figure S1**). Sample correlation was calculated using the Pearson correlation coefficient between FPKM values of both the above samples, and an acceptable correlation value of 0.84 was obtained, which was used for DEG analysis by Cuffdiff (Trapnell et al., 2012). To enhance the analysis accuracy, multi-read-correction and frag-bias-correct options were applied. All other options were set to default values. DEGs were identified based on a q-value threshold of less than 0.05 for correcting errors caused by multiple testing (Benjamini and Hochberg, 1995).

#### Gene ontology analysis

GO analysis was carried out according to biological processes of genes that are overrepresented among significantly upregulated and downregulated genes using Revigo webtool (http://revigo.irb.hr). Revigo summarizes GO terms into clusters based on their semantic similarity measures (Supek et al., 2011). Significant GO terms were clustered together into representative subsets on scatter plots where the size of the bubbles indicate generality or frequency of the GO term, while bubble colour indicates log10 of the *p*-value.

#### KEGG pathway analysis

The *E. coli* KEGG pathway data containing unique gene IDs were gathered for alignment. Genes obtained from DEGs were aligned with the acquired KEGG pathway gene IDs manually. Finally, colours ranging from green to red were designated based on log2-fold changes in the DEGs between control and the case, representing from positive to negative values respectively.

### sRNA-seq analysis

To separate and enrich regulatory RNAs from total RNA transcripts, libraries were prepared for 50 bp single-end sequencing using the NEXTflex small RNA-seq kit (Bioo Scientific Corp.). Briefly, 1 μg of total RNA molecules were ligated sequentially with 3’ and 5’ adaptors. The ligated RNAs were synthesized as single-stranded cDNAs by reverse transcription priming. By applying this as a template for second strand synthesis, double-stranded cDNA was prepared by PCR. To separate cDNA derived from sRNA, the fragments around 150 bp were isolated by gel electrophoresis and extracted for sequencing. To enrich the separated cDNA library, the products were amplified using PCR. The quality of amplified cDNA library was evaluated with an Agilent 2100 BioAnalyzer (Agilent, CA, USA), followed by quantification with the KAPA library quantification kit (Kapa Biosystems, MA, USA). Following cluster amplification of denatured templates, single-end (50 bp) sequencing was conducted on an Illumina HiSeq2500 (Illumina, CA, USA). Eighty non-coding transcripts, including sRNAs, tRNAs, antisense sRNAs and antitoxin RNAs were mapped, and sRNAs were plotted according to their Fragments Per Kilobase of transcript per million (FPKM) values. To obtain names and classes of different regulatory RNAs, transcripts were mapped to an RNA family database (Rfam) (Nawrocki et al., 2015).

### Reverse transcription and qRT-PCR

Total RNA extraction from wild-type BW25113 was performed as mentioned above. A SuperiorScript III Reverse Transcriptase cDNA synthesis kit (Enzynomics, Daejeon, Korea) was used. Relative transcript levels were determined using cDNA synthesized from 1 μg of RNA. Primer sets for target genes were designed using Primer3 (Untergasser et al., 2012). The efficiency of PCR amplification of each gene was determined using the slope of a standard curve constructed using 10-fold diluted cDNA samples. Efficiency is calculated by the following equation: Efficiency = (10^[-1/slope]^-1)×100. The primers used for qRT-PCR, their efficiency, and regression coefficient (R^2^) are listed in **Supplementary Table S4**. qRT-PCR was performed on a CFX-Connect Real-Time platform (Bio-Rad) with each 10 μl reaction a mixture comprising 2 μl of diluted cDNA (1:200), 0.5 μl of each primer (10 pM) and 5 μl of 2x iQ SYBR Green Supermix (Bio-Rad). The cycling conditions were as follows: 95°C for 5 min, followed by 40 cycles of 95°C for 20 s, 55°C for 30 s, and 72°C for 30 s. At the end of the process, the temperature was raised from 60°C to 95°C to obtain the dissociation curve. Relative expression of target genes was determined using the 2^-ΔΔCt^ method, with the 16S rRNA gene used for normalization. Relative expression was estimated by comparing the mRNA levels of each gene indicated in the figure. All experiments were performed in triplicate.

### Biomass and dissolved oxygen measurement

The Biolector I microbioreactor system was used for biomass (scattered light) dissolved oxygen tension (DOT) measurement (Samorski et al., 2005). Overnight cultures of *E. coli* MG16555 or the *ΔfnrS* mutant were diluted to an OD_600_ value of 0.05 and allowed to grow in fresh M9 minimal media supplemented with 0.2% casamino acids and 10 mM glucose until the OD_600_ value reached 0.3. Cells were treated with the desired concentration of antibiotic, and 1 ml of the treated cultures was added to an MTP-48-BOH flower plate (m2p labs) embedded with oxygen and pH-sensing optodes. During course of the run, shaking speed was set to 400 rpm, with 85% humidity at 37°C. Measurements were made in real time by setting the read cycle time at every 10 min for 24 h. Data were measured using the offline data editor option in Biolection software version 2.4.1.0 (Biolector^®^, m2p-labs GmbH, Baesweiler, Germany).

### Fluorescence dye-based ROS detection assay

Fluorescent-dye indicator 2´, 7´-Dichlorofluorescin diacetate (H2DCFDA) was purchased from Sigma. Overnight grown cultures of *E. coli* MG16555 or MG1655 *ΔfnrS* mutant were diluted to OD_600_ value of 0.05 and were allowed grow in 25 ml of LB media until OD_600_ value of 0.5. Cells were washed with phosphate saline buffer (PBS), followed by centrifugation, and resuspended in PBS. H2DCFDA (1 µM) was added to each aliquot of 3 ml, followed immediately by polymyxin B (Pol B; 0.5 µg/ml), ampicillin (10 µg/ml), cephalothin (20 µg/ml), and ciprofloxacin (10 ng/ml). A total of 100 µl of each aliquot mix were loaded onto 96-well black microplates in triplicates and the fluorescence was measured using Tristar2 LB 942 multimode reader.

## Results

### Expression of genes related to anaerobic respiration is upregulated in response to antibiotic treatment

**Figure 1:**
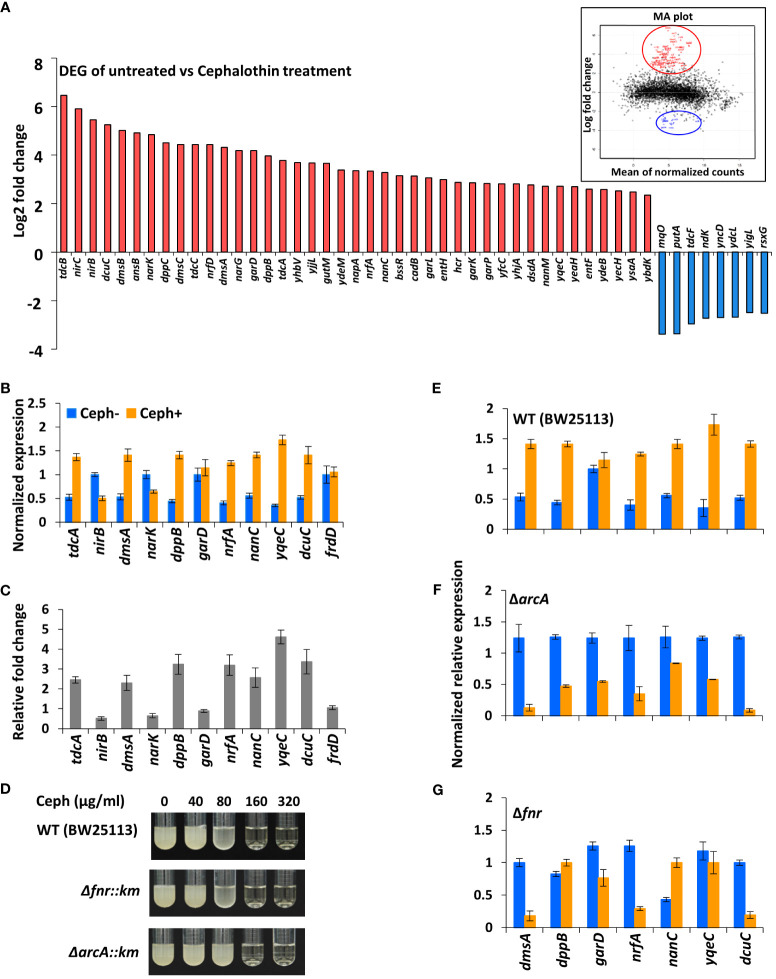
Antibiotics induce genes involved in anaerobic respiration. **(A)** DEG analysis of RNA-seq data from untreated and cephalothin-treated *E*. *coli*. Genes were up and down regulated in response to cephalothin treatment (40 µg/ml) as indicated by log_2_ fold change. Inset shows MA plot distributions of the same results. **(B)** Validation of mRNA levels of the representative genes from nine upregulated operons by qRT-PCR in untreated (Ceph-) and cephalothin-treated (Ceph+) BW25113. Expression of every mRNA was normalized to that of 16S rRNA, and relative expression was calculated by comparison with *garD* mRNA in the cephalothin-untreated wild-type. Data are presented as the normalized expression values of representative upregulated genes. Means and standard deviations are calculated from three independent experiments. **(C)** Relative fold changes in mRNA levels in cephalothin-treated cells compared with that in untreated cells were calculated using the values in **(B)**. **(D)** MIC levels of cephalothin in the indicated strain backgrounds were determined with 0 to 320 µg/ml of cephalothin. Experiments were performed in triplicate. Representative data are shown. **(E–G)** Normalized expression values of the representative genes from 9 upregulated operons determined by qRT-PCR in wild-type (BW25113), the *Δfnr* mutant and the *ΔarcA* mutant strains, respectively. Expression of every mRNA was normalized to that of 16S rRNA, and relative expression was calculated by comparison with *garD* mRNA in the cephalothin-untreated wild-type. Data are presented as the normalized expression values of representative upregulated genes. Means and standard deviations are calculated from three independent experiments.

First-generation cephalosporins are active primarily against Gram-positive bacteria. To study the early responses of Gram-negative bacteria on exposure to moderately effective antibiotics and to understand the influence of antibiotic-induced global transcriptional responses, total RNA-seq was performed using *E. coli* MG1655 treated with a sublethal concentration 40 µg/ml of cephalothin on an Illumina HiSeq 2500 platform (**Supplementary Figure S1**). A total of 223 genes were differentially expressed (*P_adj_* value ≤ 0.05), of which 157 genes were upregulated and 66 were downregulated in response to antibiotic treatment. Visual representation of the transcriptome data using an MA plot with a cut-off value ≥ 2-fold change in expression and a *P*_adj_ value ≤ 0.05 revealed 41 upregulated and eight downregulated genes (**Figure 1A**, **Supplementary Table S1**). Among the DEGs, a unique set of 23 genes embedded in nine operons – *tdcABC*, *nirBDC*, *dmsABC*, *narGHJI*, *dppABCDE*, *garPLRK*, *nrf*, *nanC* and *yqeBC* were highly upregulated, most of which are known to be involved in anaerobic respiration and in maintaining redox state of the cell (**Supplementary Figure S2**). Two different classes of transporter systems and a voltage-dependent outer membrane channel were among the upregulated sets of operons: *dppABCDE*, an ATP-binding cassette (ABC) transporter; *garPLRK*, a major facilitator superfamily (MFS) transporter; and *nanC*, an *OmpG* porin. As expected, transcript levels of representative genes (*dmsA*, *dppB*, *garD*, *nrfA*, *nanC*, *yqeC*, and *dcuC*) from operons were highly upregulated under antibiotic treatment conditions (**Figures 1B, C**). When the threshold of *P*_adj_ value was lowered (*P*_adj_ ≤ 0.01) without 2-fold change criterion, additional 59 genes (**Supplementary Figure S3A**) from seven operons, *aceEF*, *atpIBEFHAGDC*, *cyoABCDE*, *dadAX*, *pyrBI*, *rsxABCDGE*, and *sdhCDAB*, which together orchestrate the TCA cycle, proton translocation, and ATP synthesis were downregulated (**Supplementary Figure S3B**).

### *E. coli* undergoes a shift to anaerobic respiration in an FNR- and ArcA-dependent manner

Computational analysis of the promoter upstream regions of nine upregulated operons using the EcoCyc database (https://ecocyc.org) suggested exclusive binding sites and regulation by FNR and ArcA, the global regulators of anaerobic respiration pathways that mediate downregulation of the components of TCA cycle and the oxidative electron transport system (Compan and Touati, 1994; Park et al., 2013). To test the functional role of FNR and/or ArcA in antibiotic tolerance, Δ*fnr* and Δ*arcA* mutants of the wild-type strain were tested for MIC levels. The Δ*fnr* and Δ*arcA* mutants did not display notable difference in MIC levels compared to wild-type *E. coli* cells (**Figure 1D**), indicating the existence of a compensatory mechanism to induce tolerance to antibiotic stress. However, real-time qPCR of cells exposed to 40 µg/ml of cephalothin showed decrease in transcript levels of representative genes in the Δ*fnr* mutant except for *dppB* and *nanC*, with dramatic decrease in transcript levels in the Δ*arcA* mutant (**Figures 1E–G**). Taken together, our results suggest that FNR and ArcA orchestrate a sudden shift from aerobic respiration to anaerobic adaptation, triggering the redox state in the cell as an immediate response to antibiotic stress.

**Figure 2:**
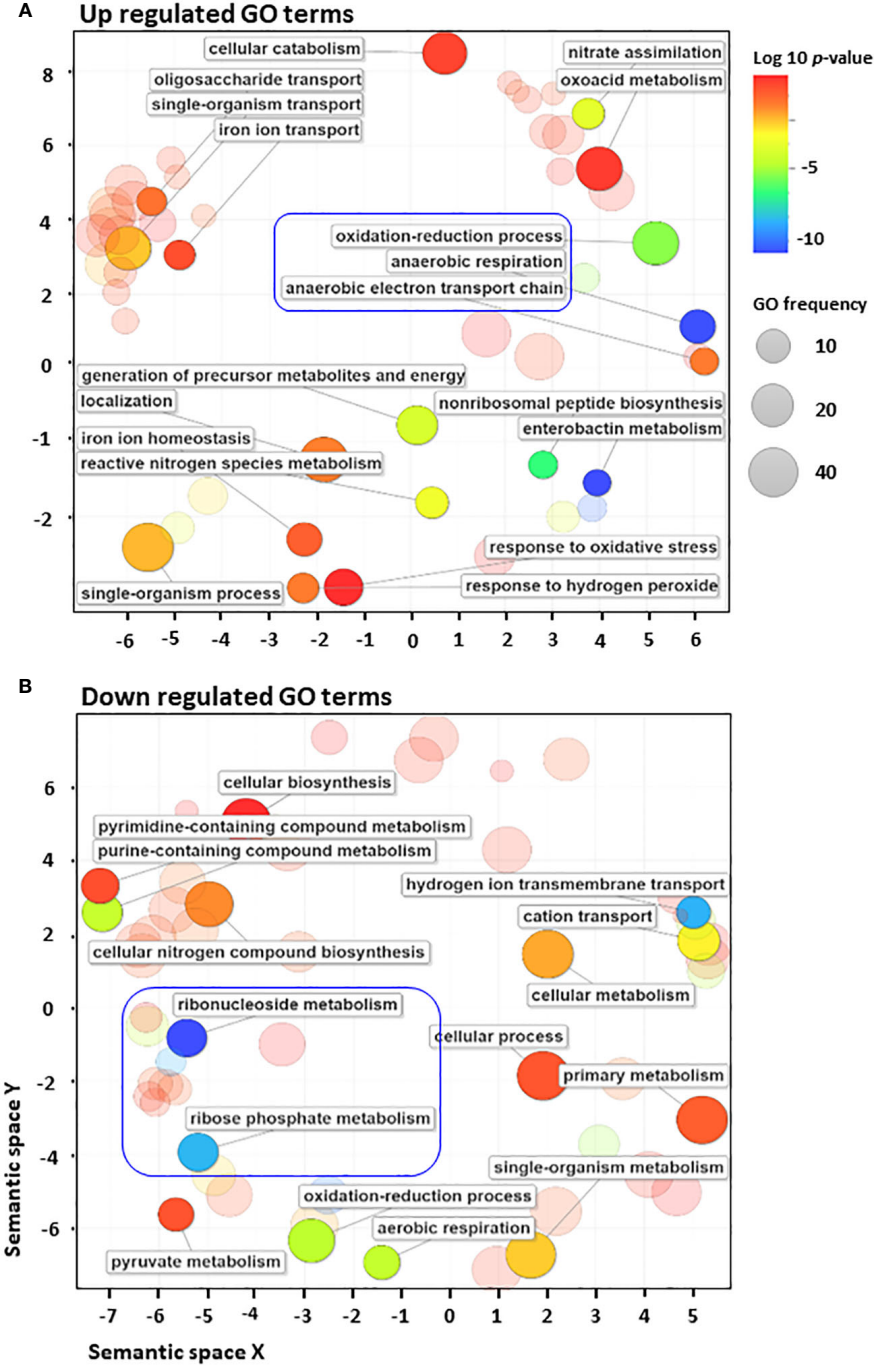
*E*. *coli* converts to anaerobic respiration when exposed to antibiotics. **(A, B)** Gene Ontology (GO) enrichment analysis of highly upregulated **(A)** and downregulated **(B)** GO terms using the Revigo web tool, which is based on semantic similarity measures. The size of the bubbles indicates the frequency of the GO term. For example, the bigger the bubble, the more common the GO term. To complement the frequency distribution bubble indicators, the bubble colour indicates the log_10_ of *p*-value.

To further analyse the distribution of 223 differentially regulated genes, biological processes from various GO terms were grouped based on their semantic similarity measures (Fantappiè et al., 2011) (**Figure 2**). Among GO terms of upregulated genes, those related to anaerobic respiration and enterobactin metabolism were the most enriched, as indicated by the blue colour (**Supplementary Figure S4A**, **Supplementary Table S2**). By contrast, many GO terms of downregulated genes are associated with various biosynthetic pathways, aerobic respiration, cellular metabolism and cation transport (**Supplementary Figure S4B**, **Supplementary Table S2**). Significant GO terms were clustered together into representative subsets on the scatter plots, in which the size of the bubbles indicates the frequency of the GO term. The frequency distribution of GO terms, as indicated by the size of the bubbles, was larger among downregulated terms (in sharp contrast to upregulated terms), indicating that highly specific genes were upregulated in response to antibiotic stress (**Figure 2**). Moreover, GO terms related to anaerobic respiration and enterobactin metabolism were highly upregulated, whereas those related to ribonucleoside and ribophosphate metabolism were highly downregulated (**Figure 2**). To explore the metabolic perturbations at the pathway level, KEGG pathway analysis was performed by comparing untreated vs. cephalothin-treated cells (**Supplementary Figures S5–S11**). Metabolic pathways related to the TCA cycle, oxidative phosphorylation, aerobic β-oxidation and ribosome synthesis were highly downregulated, as shown by the colour bar indicator in **Supplementary Figures S4–S6**. Conversely, pathways specific to anaerobic adaptation such as galactose metabolism, glycolysis, gluconeogenesis, phosphotransferase system, non-ribosomal peptide biosynthesis, and nitrogen metabolism were upregulated (**Supplementary Figures S7–S9A**). Mechanistic pathways related to flagellar assembly and chemotaxis were also upregulated (**Supplementary Figures S9B, S10**).

### ArcA and FNR regulate expression of sRNAs FnrS and Tp2

**Figure 3:**
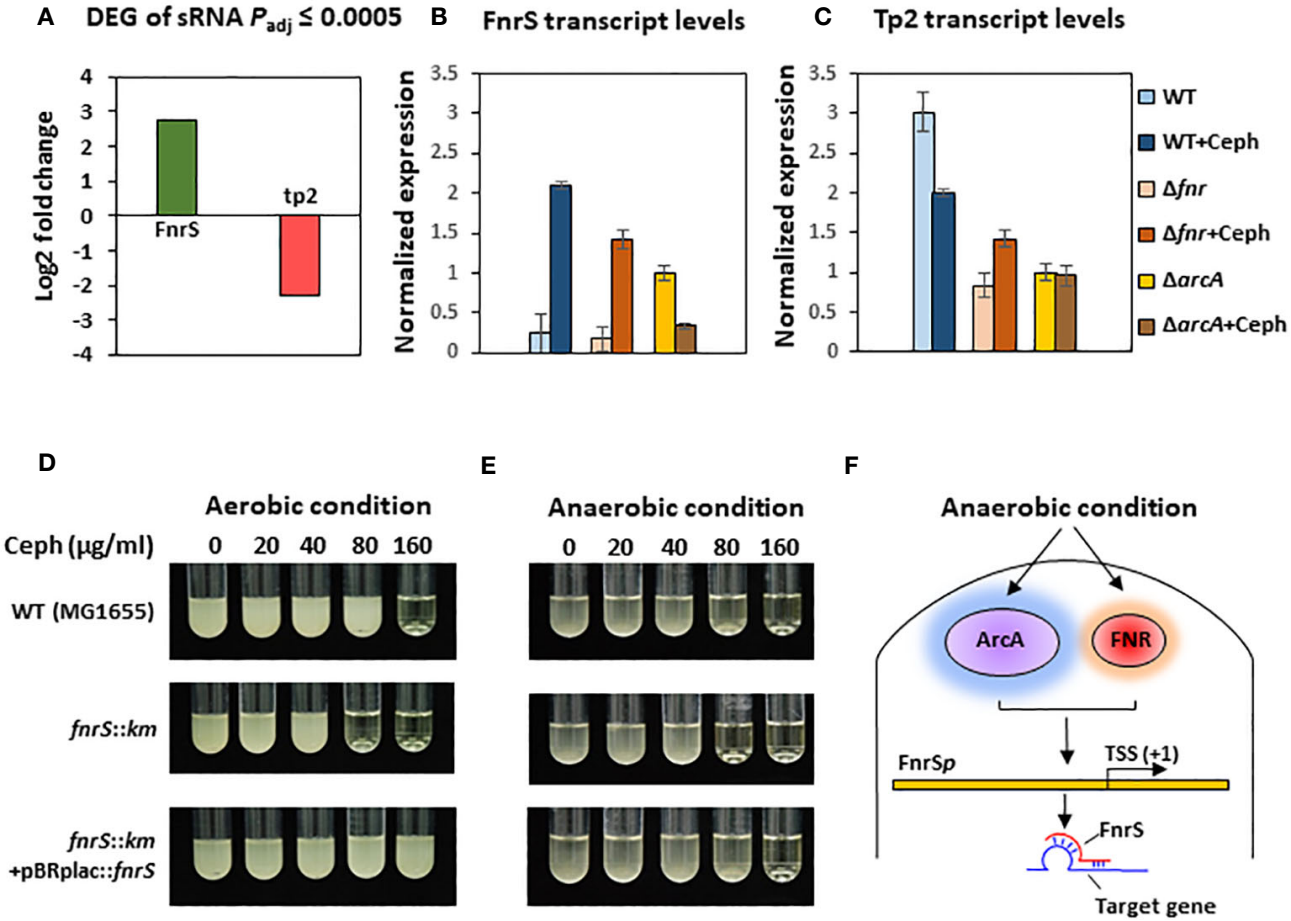
Small RNA FnrS and Tp2 are regulated by antibiotic exposure. **(A)** Relative sRNA expression between the untreated and cephalothin-treated sample depicted by Log_2_ fold change of DEG of sRNAs (*p*_adj_ = 0.0005) upon 40 µg/ml cephalothin treatment. **(B, C)** The sRNA expression was validated using qRT-PCR. Transcript levels of FnrS **(B)** and Tp2 **(C)** sRNA were compared under indicated strain backgrounds that were treated with 40 µg/ml cephalothin. In both graphs, sRNA levels in each strain were normalized to those of 16S rRNA, and relative expression was calculated by comparison with the sRNA level in the cephalothin-untreated *arcA* mutant. Means and standard deviations are calculated from three independent experiments. **(D, E)** MIC levels of wild-type (MG1655), the *ΔfnrS* mutant and the complemented strain (*ΔfnrS+*pBRplac::*fnrS*) treated with 0 to 160 µg/ml of cephalothin under aerobic **(D)** and anaerobic conditions **(E)**. Experiments were performed in triplicate. Representative data are shown. **(F)** Diagrammatic representation of putative mode of FnrS regulation by ArcA and FNR under anaerobic conditions. FnrS is expressed under anaerobiosis and downregulate target genes in *E*. *coli*.

To further investigate the mechanisms underlying the post-transcriptional regulation by the non-coding region, we conducted sRNA-sequencing (sRNA-seq) to compare the sRNA transcriptomes of cephalothin-treated and untreated bacterial cells. Data analysis showed alleviated expression of sRNAs such as RyhB and CsrB, which are involved in the iron starvation response and central carbon flux, respectively, indicating the effects of cephalothin on central cellular processes (Massé and Gottesman, 2002; Babitzke and Romeo, 2007; Urbanowski et al., 2000) (**Supplementary Figure S11**, **Supplementary Table S3**). Analysis of DEG sRNAs (*P*_adj_ ≤ 0.0005) with respect to the log_2_ fold expression change revealed FnrS and Tp2 as two uniquely expressed sRNAs with a 2.7 log_2_-fold increase and 2.3 log_2_-fold decrease in expression, respectively (**Figure 3A**). Relative transcript levels were verified by qRT-PCR (**Figures 3B, C**). To test the functional role of FnrS in antibiotic stress, the MIC levels of cephalothin were determined in *E. coli* strains under aerobic and anaerobic conditions. As expected, the mutation of *fnrS* gene decreased the MIC level of cephalothin under both aerobic and anaerobic conditions, and those decreases were complemented with the *fnrS* expression by the pBRplac::FnrS plasmid (**Figures 3D, E**; **Supplementary Figure S12**). Both FNR and ArcA orchestrate complex regulatory interplay and co-regulate FnrS that targets multiple genes involved in oxidative metabolism by direct base pairing with their mRNAs, leading to translation repression (Boysen et al., 2010; Durand and Storz, 2010; Fantappiè et al., 2011; Park et al., 2013) (**Figure 3F**).

FnrS is strictly expressed under anaerobic conditions, while Tp2 is predicted to regulate the 50S ribosomal subunit L23 (Boysen et al., 2010; Durand and Storz, 2010). DEG analysis revealed a marked downregulation of *mqo*, *sdhA, dadA*, *iscR*, and *atpF* and upregulation of *yhbV* and *nirB* which are either predicted targets of FnrS through CopraRNA webpage or experimentally demonstrated in previous reports (**Table 1**). In addition, downregulation of Tp2 correlates with levels of L23 transcripts and the ribosome assembly KEGG pathway (**Supplementary Figure S5B**). Taken together, the sRNA-seq results show that the sRNAs FnrS and Tp2 are differentially expressed when bacterial cells are exposed to antibiotics. Moreover, downstream regulation by the two sRNAs correlates with the results of RNA-seq, suggesting that bacteria regulate genes to adapt to the toxic, antibiotic-rich environment by perturbing cellular respiration and protein synthesis pathways.

**Table 1:** The differentially expressed genes and their GO terms obtained from RNA-seq analysis that are putative targets of FnrS and Tp2.

sRNA	Target Gene	Description	GO term (Biological process)	Log2 fold change
FnrS	*mqo*	malate dehydrogenase, FAD/NAD(P)-binding domain	TCA cycle	-3.38
	*sdhA*	succinate dehydrogenase, flavoprotein subunit	TCA cycle	-2.93
	*dadA*	D-amino acid dehydrogenase	Oxidation-reduction process	-2.45
	*iscR*	DNA-binding transcriptional repressor	Transcription regulation	-2.31
	*atpF*	F0 sector of membrane-bound ATP synthase, subunit b	ATP biosynthesis	-1.87
	*yhbV*	putative protease	Proteolysis	3.69
	*nirB*	nitrite reductase, large subunit, NAD(P)H-binding	Anaerobic respiration	5.46
Tp2	*rpIW*	50S ribosomal subunit protein L23	translation	-1.95

### ROS levels are regulated by FnrS in response to treatment with antibiotics

**Figure 4:**
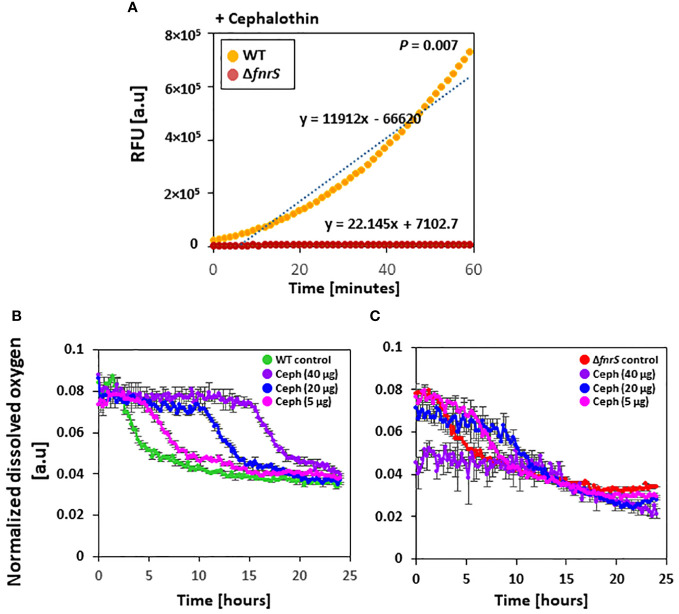
FnrS is responsible for ROS production and delays oxygen uptake. **(A)** Fluorescence-based ROS detection was observed in wild-type and the *ΔfnrS* mutant. Bacterial cells were grown to OD_600_ value of 0.4 and treated with 20 µg/ml cephalothin. Then, 1 mM of ROS fluorescein dye H2DCFDA was immediately added. Relative fluorescence units (RFU) were determined using a fluorimeter. As ROS increased over 60 minutes, trend lines were plotted on the graph. The slopes of these trend lines were compared to determine differences. The experiment was performed in triplicate, and a representative figure is shown. *P*-value was determined with two-tailed Student’s *t*-test. **(B, C)** Dissolved oxygen levels in bacterial culture medium from untreated and cephalothin-treated samples (5, 20, and 40 µg/ml) were measured. Cells were grown to OD_600_ of 0.5 and treated with indicated amounts of cephalothin (µg/ml) at 37°C using wild-type **(B)** and *ΔfnrS* mutant strains **(C)**. Normalized dissolved oxygen levels were obtained by dividing the oxygen levels in culture medium by the biomass value. All experiments were performed in triplicate. Representative data are shown.

Antibiotics are reported to induce oxidative stress and elevate the cellular redox state (Belenky et al., 2015). However, the role of ROS in antibiotic-mediated cell death remains contradictory (Kohanski et al., 2007; Keren et al., 2013; Liu and Imlay, 2013). To assess the role of FnrS in ROS production, fluorescence-based ROS detection assays (Dong et al., 2015) were performed in which wild-type and the Δ*fnrS* mutant were exposed to four different antibiotics: cephalothin, polymyxin B, ciprofloxacin, and ampicillin (**Figure 4A**, **Supplementary Figure S13**). Sublethal concentrations that generate ROS at comparable levels to those in untreated wild-type cells were used. ROS production was identical in wild-type and the Δ*fnrS* mutant unexposed to antibiotics (**Supplementary Figure S14A**). However, ROS production was significantly reduced in the Δ*fnrS* mutant compared to wild-type cells following antibiotic exposure, regardless of the type of antibiotics (**Figure 4A**; **Supplementary Figure S13**). Interestingly, however, the decrease in ROS production in the Δ*fnrS* mutant was antibiotic class-specific. In the case of polymyxin B, decrease in fluorescence of 1.2-fold was observed compared to that of wild-type cells (**Supplementary Figure S13A**), while the fluoroquinolone ciprofloxacin yielded a 1.1-fold decrease in fluorescence in the Δ*fnrS* mutant (**Supplementary Figure S13B**). Strikingly, a remarkable reduction in fluorescence was seen in the Δ*fnrS* mutant treated with the β-lactam antibiotics cephalothin and ampicillin, 537-fold and 524-fold decreases, respectively (**Figure 4A**; **Supplementary Figure S13C**). These results indicate that FnrS mediates ROS production with high specificity in the case of β-lactam antibiotics.

### FnrS delays oxygen consumption upon exposure to antibiotics

Whole transcriptome analysis of the non-coding region of the genome and qRT-PCR showed that FnrS is highly activated during drug exposure (**Figures 3A, B**). To assess the role of FnrS during antibiotic exposure and oxygen uptake, the dissolved oxygen concentration in the culture media of wild-type and Δ*fnrS* mutant was measured using a real-time DOT monitor system in oxygen-limiting conditions. Cultures in 48-well flower plates fitted with oxygen and pH-sensing optodes were treated with different concentrations of cephalothin, and DOT and biomass were measured. Under untreated conditions, normalized dissolved oxygen (DOT/biomass) in both wild-type and the Δ*fnrS* mutant culture media was 0.08 at the zero time point and gradually decreased reaching 0.03 as a steady state level at the late stationary phase (**Supplementary Figure S14B**). However, at 40 μg/ml of cephalothin, normalized dissolved oxygen levels in wild-type culture medium remained at 0.08 for more than 12 h, and then fell gradually to 0.03, which is the same as the normalized dissolved oxygen level in untreated culture medium (**Figure 4B**). These results indicated minimal oxygen consumption by cells during antibiotic exposure. By contrast, Δ*fnrS* mutant culture media showed a sharp decrease of 62.5% in DOT level at the zero time point, indicating a massive uptake of oxygen by the mutant cells (**Figure 4C**). The DOT pattern in the culture media was similar but less rigorous at 20 μg/ml and 5 μg/ml cephalothin. These results indicate that FnrS is a major negative regulator of genes involved in aerobic metabolism and is vital for cell survival because it shifts cells to anaerobic adaptation during antibiotic stress.

## Discussion

Low concentrations of antibiotics exert their effects by inducing *de novo* resistance, generating genetic and phenotypic variability by mutagenesis, and acting as signalling molecules (Gullberg et al., 2011; Andersson and Hughes, 2014). Recent studies have shown that β-lactams induce metabolic fluctuations and alteration in cellular respiration that ultimately affect cell viability (Dwyer et al., 2014; Lobritz et al., 2015). Herein, we employed coding and non-coding transcriptome analysis of cephalothin-treated *E. coli* cells and identified FnrS and Tp2 as two distinctly expressed sRNAs that regulate anaerobic respiration and protein synthesis during antibiotic-associated stress.

**Figure 5:**
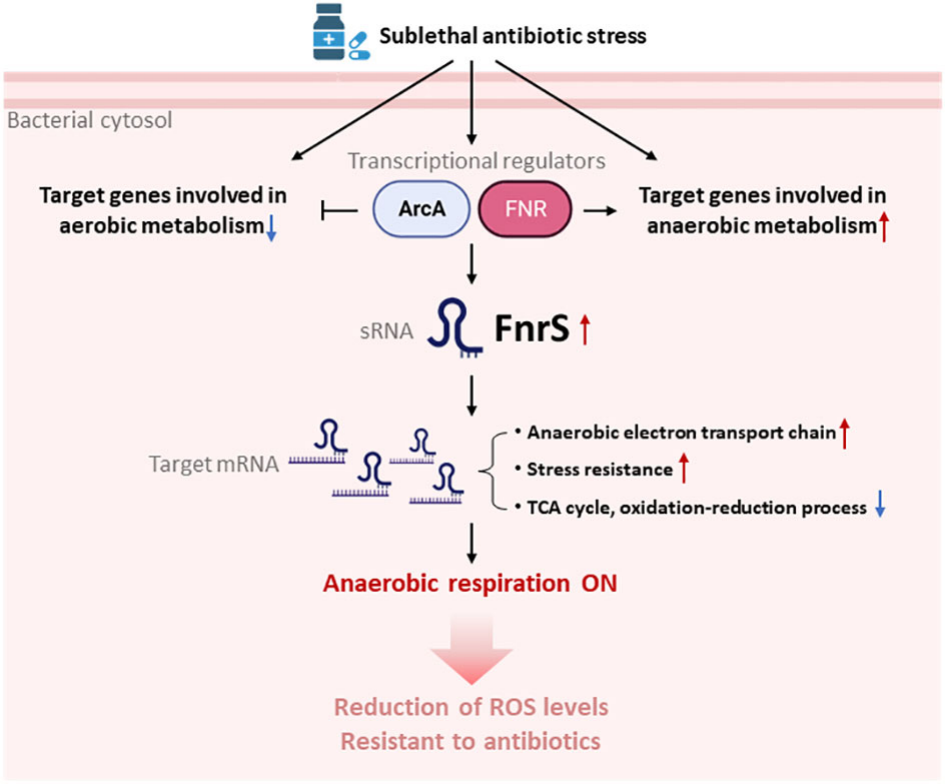
Model for the switchover to anaerobic respiration triggered by FnrS-mediated post-transcriptional regulation. Treatment with sublethal concentrations of antibiotics leads to activation of ArcA and FNR, and thus expression the sRNA FNR. Antibiotic stress also down-regulates aerobic metabolism and up-regulates anaerobic metabolism. Increased FnrS levels regulate downstream expression of target genes post-transcriptionally. Expression of genes involved in the anaerobic electron transport chain and stress resistance is increased by FnrS. Expression of genes involved in the TCA cycle and oxidation-reduction processes is decreased by FnrS. The overall switch to anaerobic respiration helps bacterial cells endure antibiotic stress. The figure was created by BioRender.com.

Our DEG analysis showed that the *cyoABCDE* and *atp* operons, encoding the cytochrome *bo* terminal oxidase complex and ATP F_1_ synthase complex, respectively, are markedly downregulated (up to 4-fold) and either could be a secondary target of β-lactam antibiotics (**Supplementary Figure S3**). In addition, studies have shown that β-lactams induce futile biosynthetic cycles of peptidoglycan synthesis and degradation (Dwyer et al., 2015). In the present work, we provide experimental evidence of respiratory changes mediated by sRNAs that enhance cell survival during antibiotic stress conditions. Our findings shed light on the mechanisms of intrinsic resistance in bacteria that have evolved to manage abrupt changes in the environment such as the release of lethal antibiotics in the surroundings by antagonistic bacteria. Here we show that antibiotics activate FNR-ArcA-FnrS circuitry in the presence of oxygen to shift cells into a less active metabolic and protective state (Lobritz et al., 2015) (**Figure 5**). Previous studies demonstrated SoxS-mediated ROS generation in bacteria exposed to a T6SS effector, P1*vir* phage, or polymyxin B (Dong et al., 2015). SoxR is activated via oxidation of its [2Fe-2S] cluster that in turn directly activates SoxS (Nunoshiba et al., 1992). Our RNA-seq data revealed a 2.5-fold decrease in the *rsxG* transcript levels, which is required for reduction of SoxR (**Supplementary Table S1**). We hypothesise that FnrS protects cellular components from antibiotic-induced ROS shock by shifting core cellular processes to anaerobic adaptation.

Lethal antimicrobial stress causes accumulation of ROS in bacterial cells (Zhao and Drlica, 2014). As a result, changes in ROS accumulation impact the lethal action of antibiotics. Previous studies demonstrate that anaerobic conditions and inhibition of ROS accumulation reduce the effects of ROS on antibiotic lethality (Zhao and Drlica, 2014). Moreover, the effects of antibiotics are partially impaired under anaerobic conditions (Keren et al., 2013). Here, we found that sublethal concentrations of antibiotics reduced ROS production by the wild-type strain (**Supplementary Figure S15**), suggesting *E. coli* may detoxify ROS in response to sublethal concentrations of antibiotics. Therefore, switching to anaerobic respiration may benefit *E. coli* by reducing ROS-induced shock triggered by antibiotics.

However, ROS production in the presence of mutated sRNA FnrS strain was lower than that by the wild-type strain after treatment with antibiotics (**Figure 4A**; **Supplementary Figure S13**). FnrS is a highly conserved and anaerobically-induced sRNA in *E. coli* (Boysen et al., 2010). FnrS regulates multiple genes, either negatively or positively, involved in central intermediary metabolism, amino acid biosynthesis, and stress resistance proteins (Boysen et al., 2010; Durand and Storz, 2010). Target genes of FnrS include *sodB*, *marA*, and *ydhD/grxD*, all of which are related to stress resistance. Among them, *sodB* encodes the superoxide dismutase enzyme (which neutralize toxic levels of ROS), and *marA* activates expression of *sodA*, another superoxide dismutase in *E. coli* (Wright et al., 2013). The reason why ROS production was reduced in the Δ*fnrS* mutant strain could be attributed to supressed expression of *sodB* and *sodA* genes by the *fnrS* mutation, thereby allowing detoxification of ROS. On the other hand, the reason why ROS production varied depending on the type of antibiotic in the Δ*fnrS* mutant remains elusive. Future work is needed to further refine the response of bacteria on other type of antibiotics.

In conclusion, our results show that antibiotic stress leads to rapid but specific reprogramming of both coding and non-coding gene transcription, which minimizes ROS levels induced by antibiotics, thereby increasing cell survival (**Figure 5**). Our study strengthens the idea of sRNAs as potential non-protein targets to develop novel antimicrobial compounds and adjuvant like substances to combat multi-drug resistance.

## Data availability statement

The datasets presented in this study can be found in online repositories. The names of the repository/repositories and accession number(s) can be found below: DDBJ, PRJDB17605.

## Author contributions

DK: Writing – review & editing, Data curation, Formal analysis, Visualization, Investigation, Methodology. AB: Writing – review & editing, Conceptualization, Investigation, Methodology, Validation, Visualization, Writing – original draft.. S-KK: Visualization, Writing – review & editing, Data curation, Software. SL: Writing – review & editing, Investigation, Methodology. C-MR: Writing – review & editing, Conceptualization, Funding acquisition, Project administration, Supervision, Writing – original draft.
