# Supplementary material for: Small RNA-modulated anaerobic respiration allows bacteria to survive under antibiotic stress conditions
Source: Front Cell Infect Microbiol. 2024 Mar 13;14:1287557. doi: 10.3389/fcimb.2024.1287557 (PMC10993149; doi:10.3389/fcimb.2024.1287557)
Supplement: Supplementary file 1 [file DataSheet_1.pdf]

Small RNA-modulated anaerobic respiration allows bacteria to survive under antibiotic stress conditions

Dajeong Kim[1†], Abhayprasad Bhat[1†], Seon-Kyu Kim[2], Soohyun Lee[1], and Choong-Min Ryu[1*]

[1]Molecular Phytobacteriology Laboratory, Infectious Disease Research Center, Korea Research Institute of Bioscience and Biotechnology, Daejeon, Republic of Korea. [2]Personalised Genomic Medicine Research Center, Korea Research Institute of Bioscience and Biotechnology, Daejeon, Republic of Korea.

*Corresponding author: Choong-Min Ryu

Tel: 82-42-879-8229

Fax: 82-42-860-4488

E-mail: cmryu@kribb.re.kr

† These authors contributed equally to this work.

Fig S1

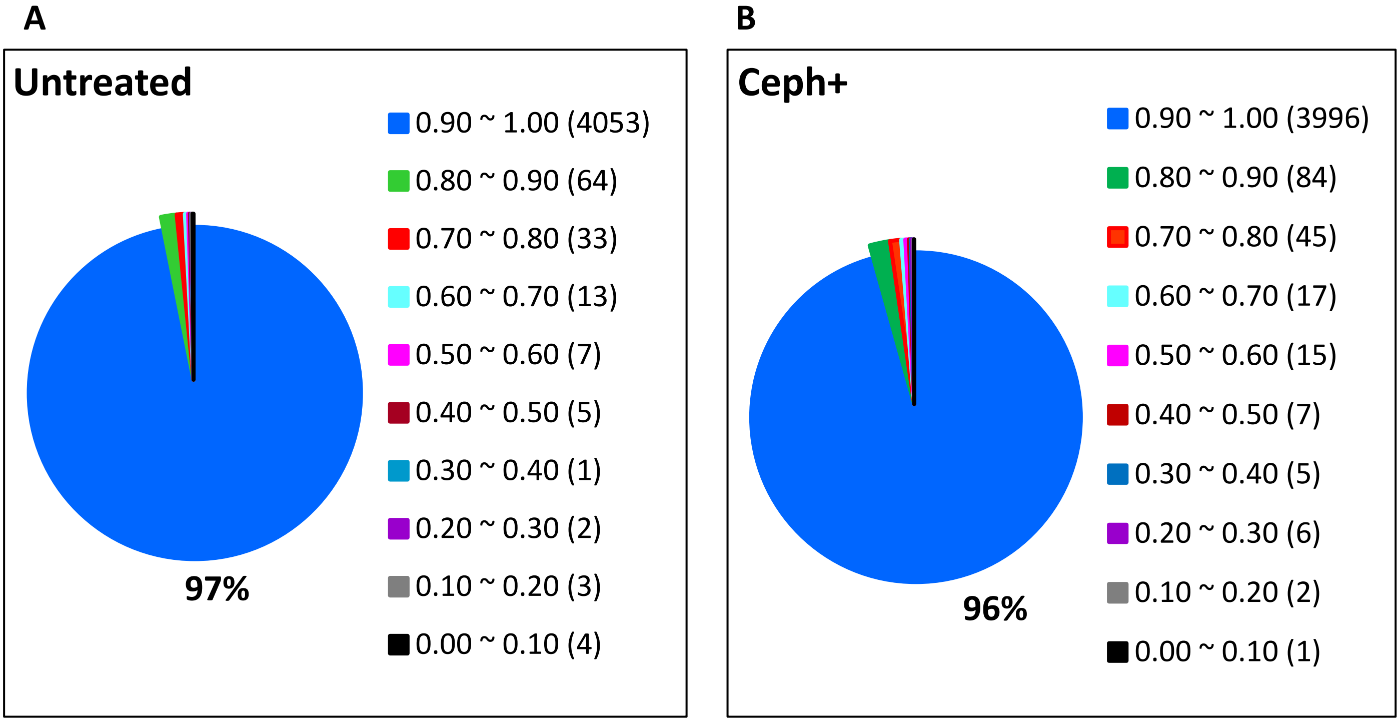

Fig. S1. Sample coverage analysis. Transcript reads were quantitated using Illumina HiSeq 2500 platform for untreated (A) and Cephalothin treated (B) samples with a reliable sample coverage of 97% and 96% respectively.

Fig S2

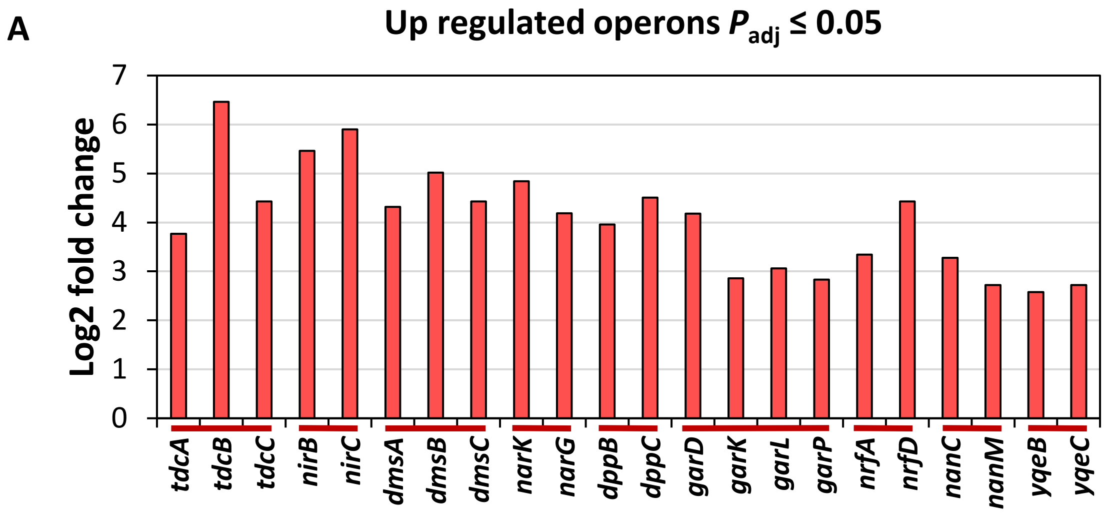

Fig. S2. Operon-specific DEG analysis of Cephalothin treated cells. (A) At P_{adj} value ≤ 0.05, nine operon-specific sets of genes were highly upregulated that are regulated by FNR and/or ArcA.

Fig S3

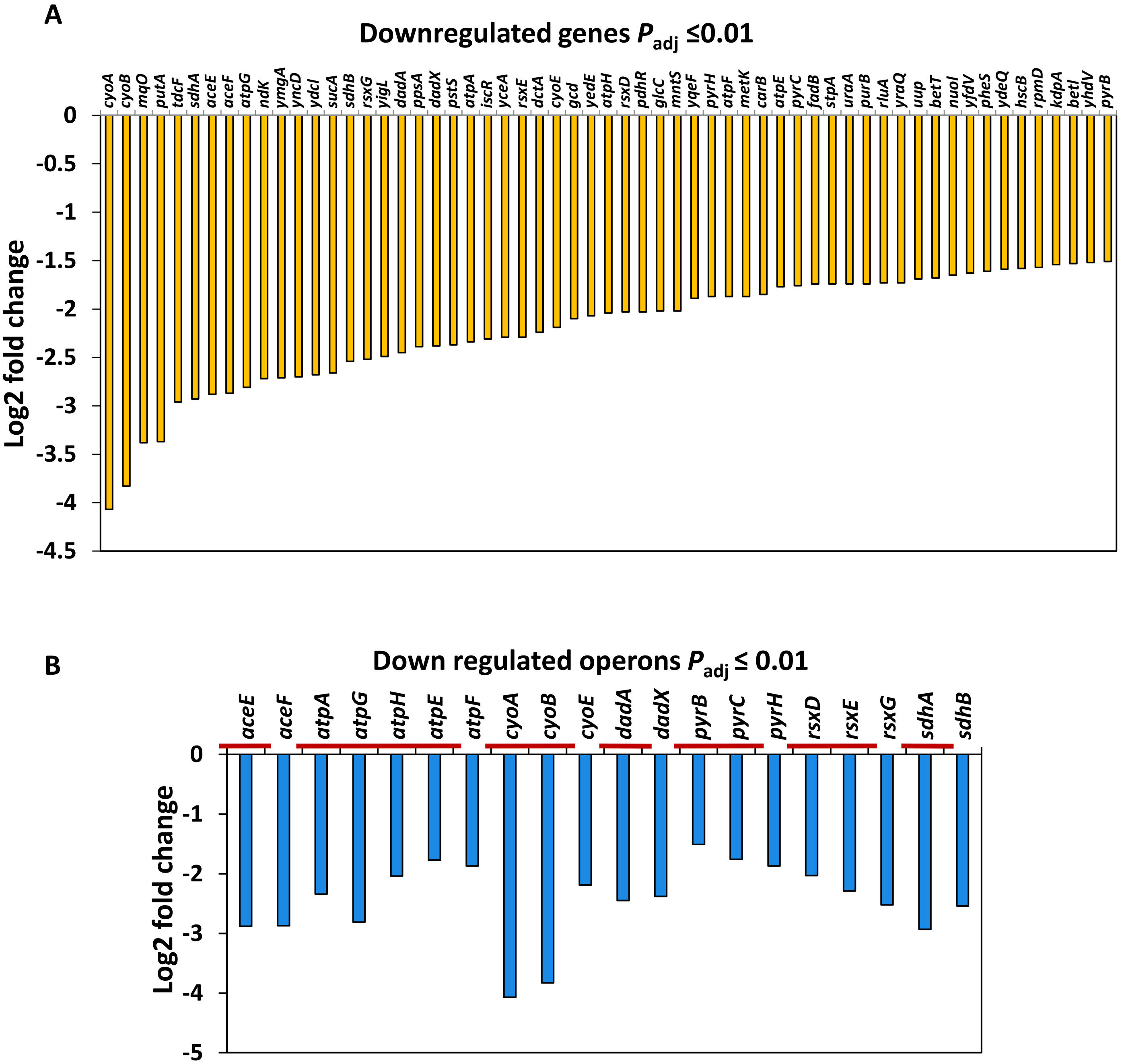

Fig. S3. DEG analysis of Cephalothin treated cells (P_{adj} value ≤0.01). (A) A total of 59 genes were downregulated in response to Cephalothin treatment (B) seven operon-specific sets of genes were downregulated.

Fig S4

KEGGG pathway analysis

Aerobic pathways (down)

A TCA cycle

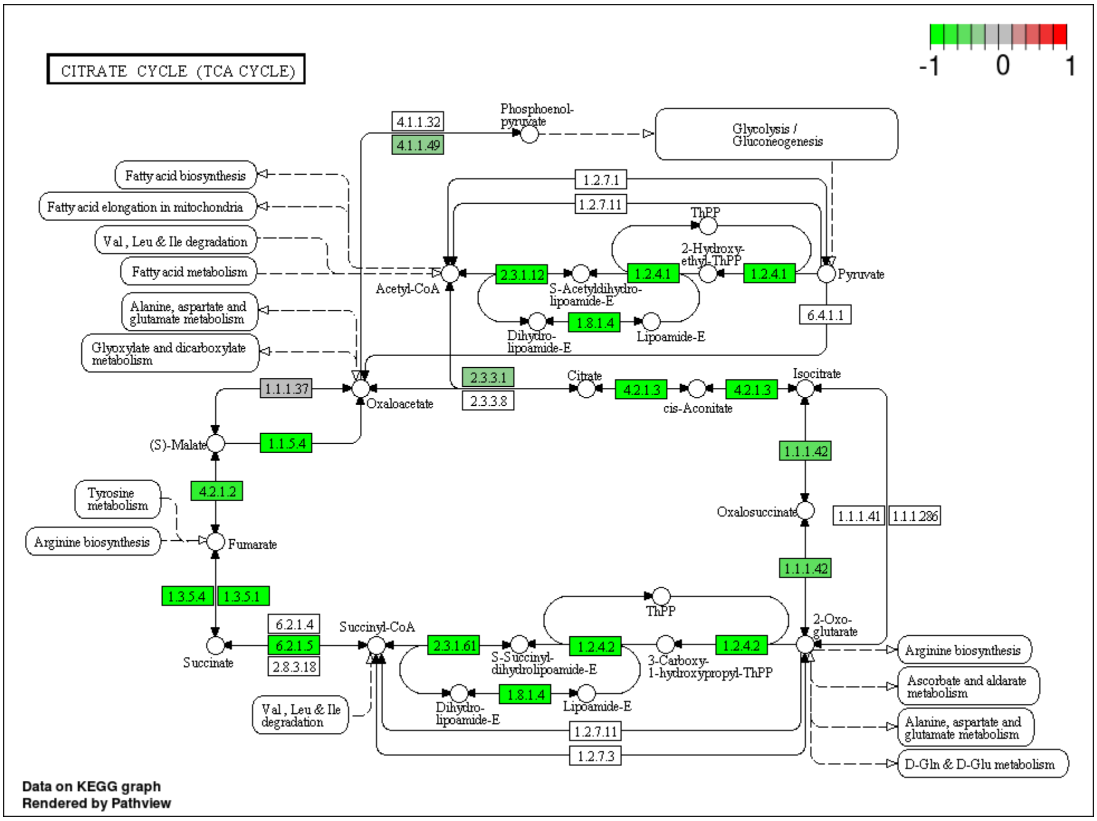

B Oxidative phosphorylation

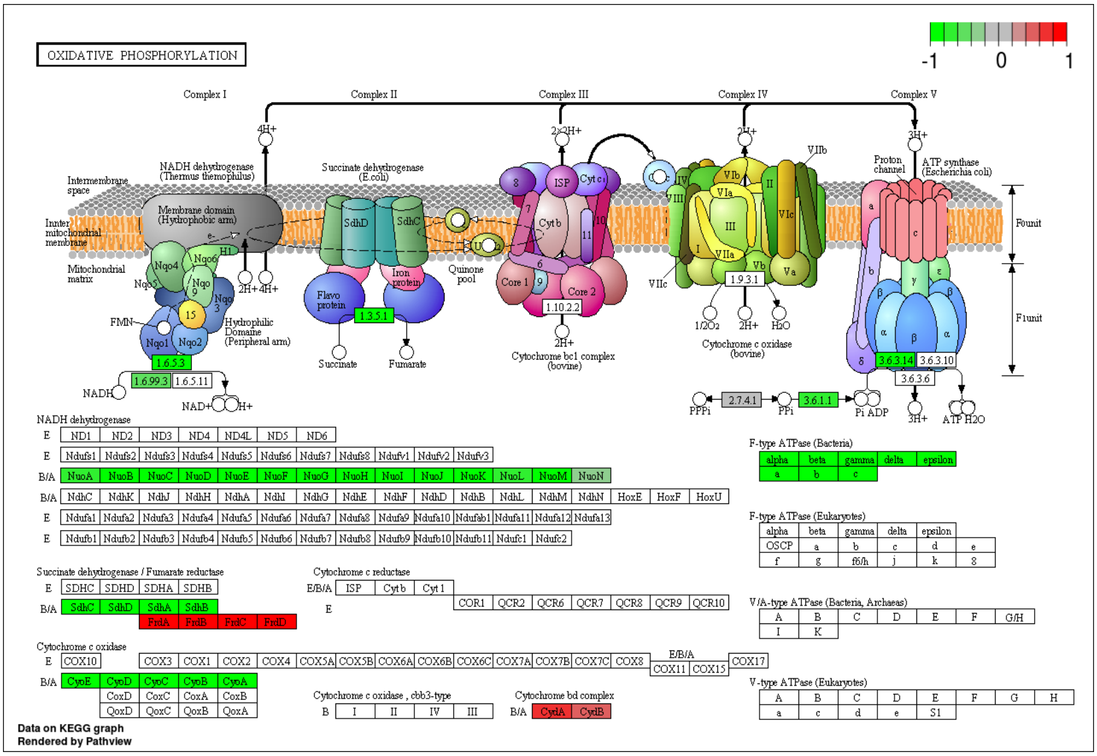

Fig S5

Aerobic pathways (down)

A **β-oxidation pathway**

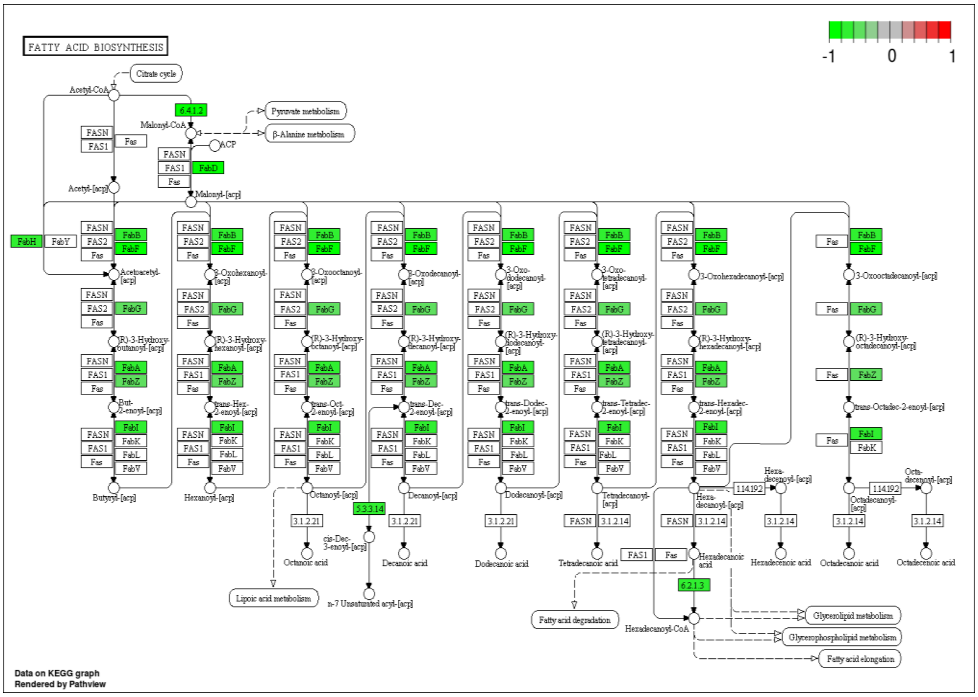

B **Ribosome assembly**

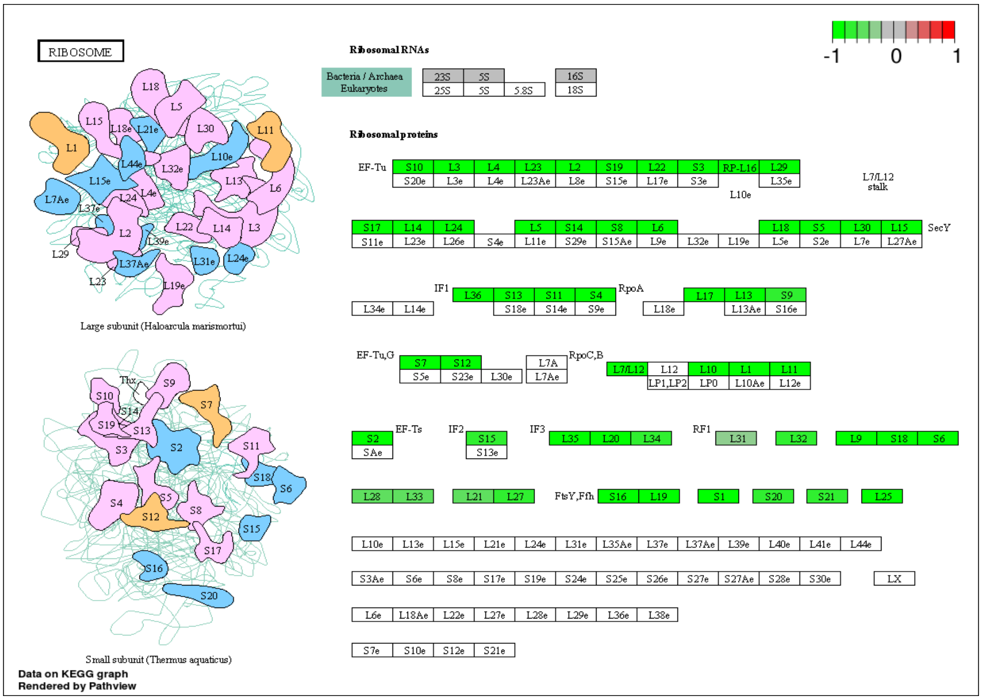

Fig. S4-5. KEGG pathway mapping of Cephalothin treated cells as determined by mRNA abundance analysis (P_{adj} value ≤0.05). (A) Significantly down regulated aerobic pathways and associated genes of β-oxidation pathway and (B) ribosome assembly. Colors that range from green to red are designated to genes extracted from log2 fold change of DEGs between untreated cells and Cephalothin treated cells.

Fig S6 Aerobic pathways (down)

A Purine metabolism

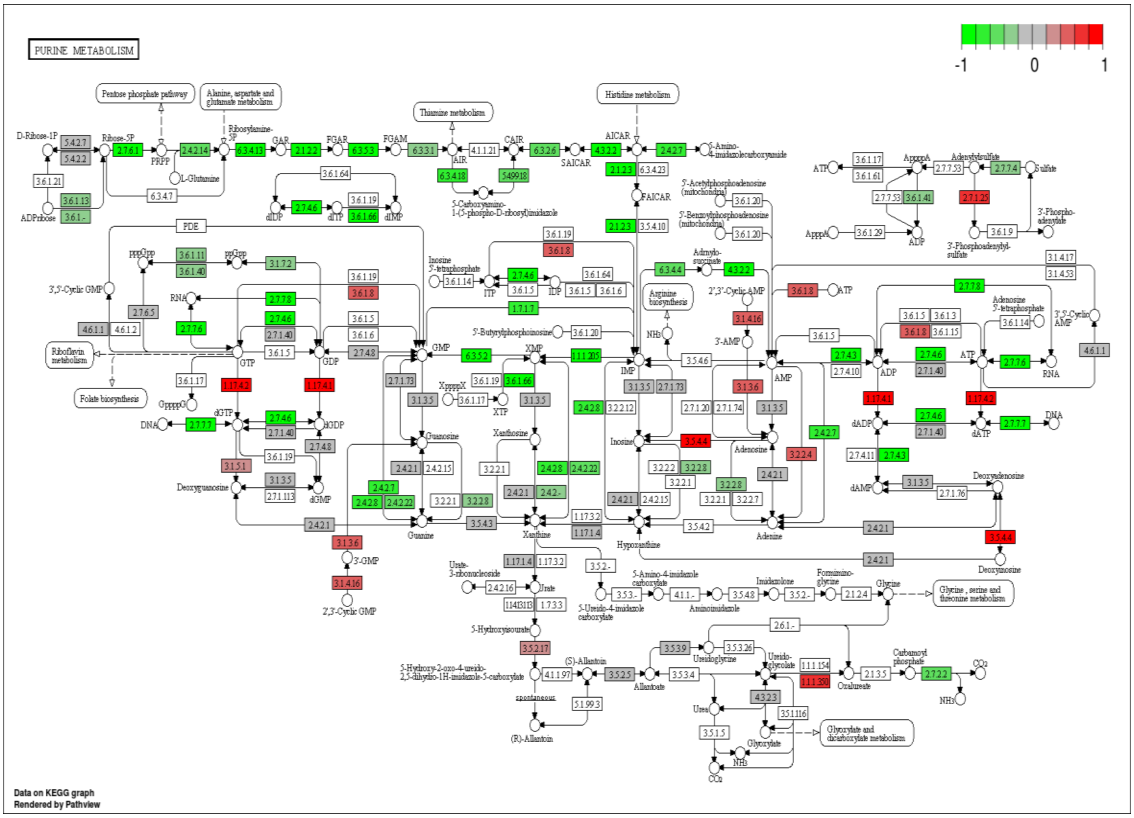

B Pyrimidine metabolism

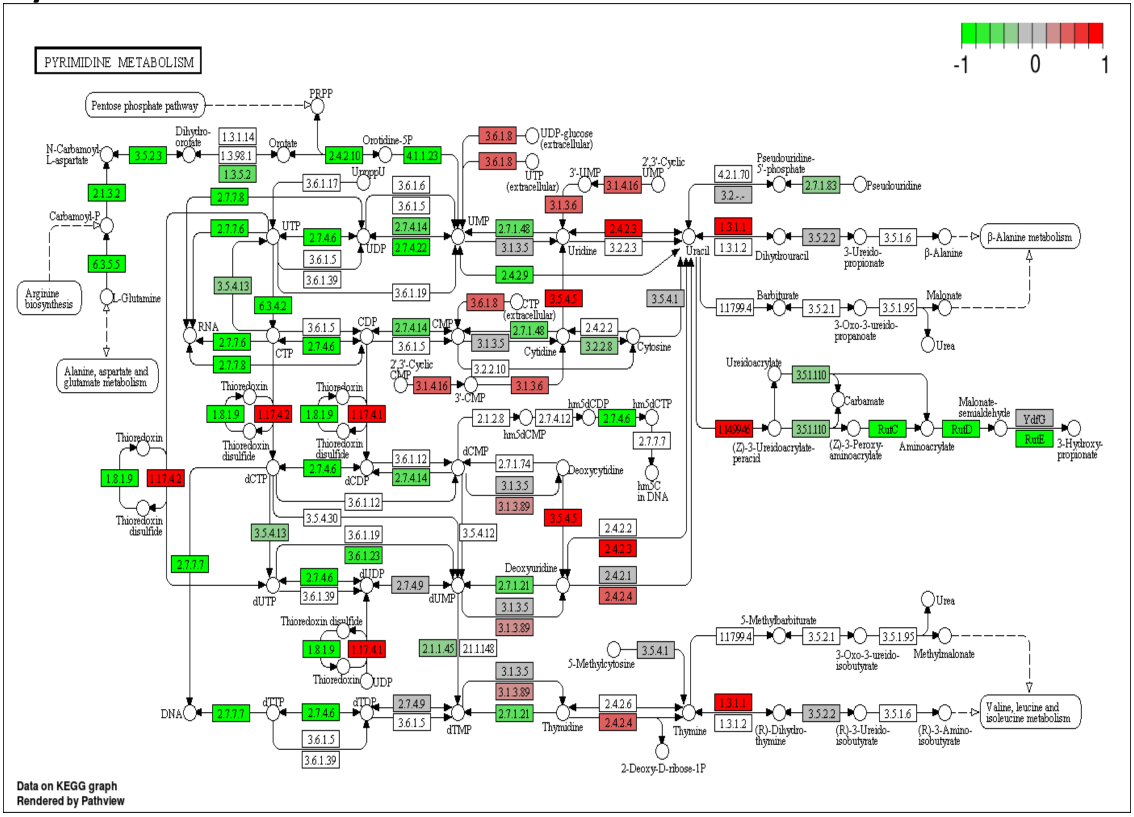

Fig. S6. KEGG pathway mapping of Cephalothin treated cells as determined by mRNA abundance analysis (P_{adj} value ≤0.05). (A) Significantly down regulated aerobic pathways and associated genes of purine metabolism and **(B)** pyrimidine metabolism. Colors that range from green to red are designated to genes extracted from log2 fold change of DEGs between untreated cells and Cephalothin treated cells.

Fig S7 Anaerobic pathways (up)

A Galactose metabolism

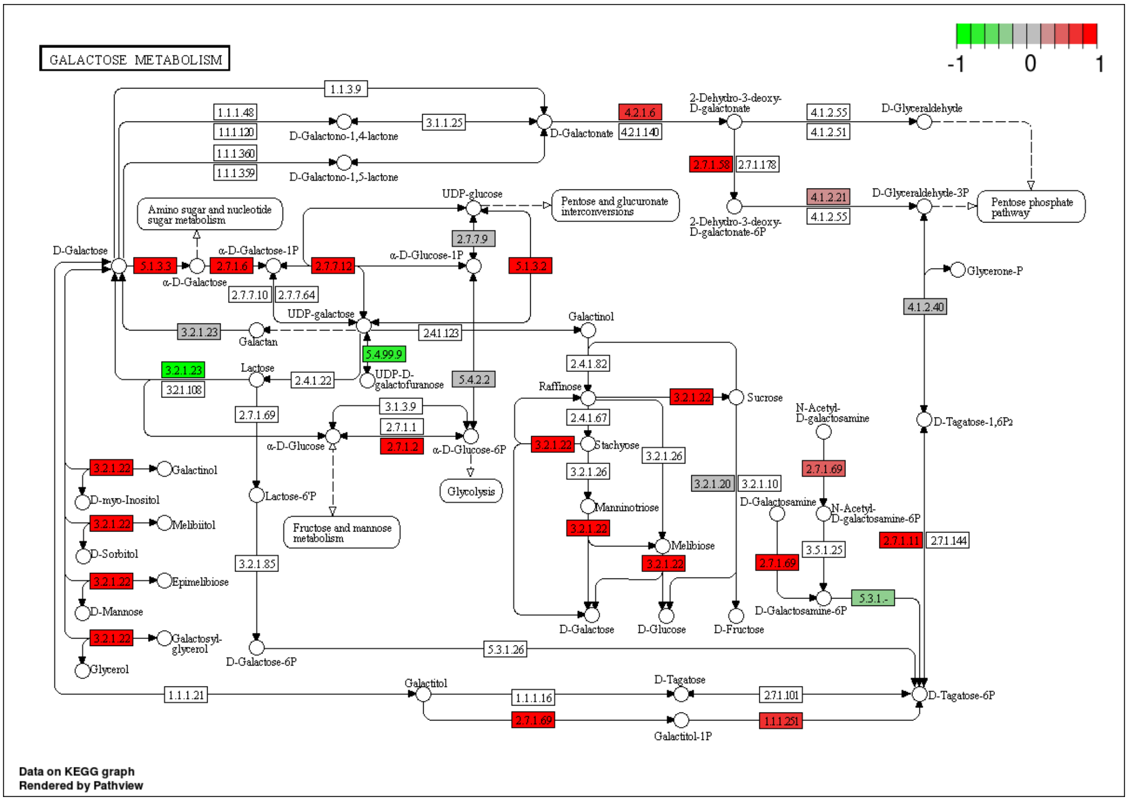

B Glycolysis/gluconeogenesis

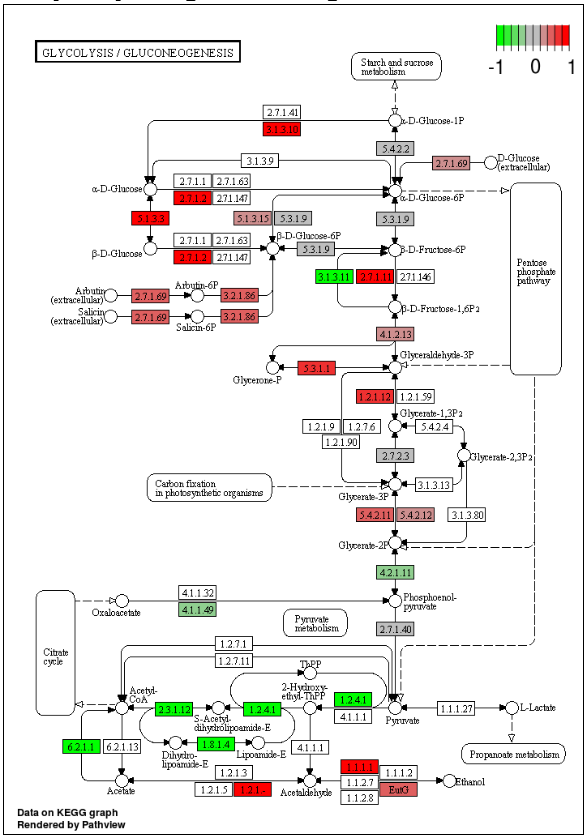

Fig. S7. KEGG pathway mapping of Cephalothin treated cells as determined by mRNA abundance analysis (P_{adj} value ≤0.05). (A) Significantly up regulated anaerobic pathways and associated genes of galactose metabolism and **(B)** glycolysis/gluconeogenesis. Colors that range from green to red are designated to genes extracted from log2 fold change of DEGs between untreated cells and Cephalothin treated cells.

Fig S8

Anaerobic pathways (up)

A Phosphotransferase system

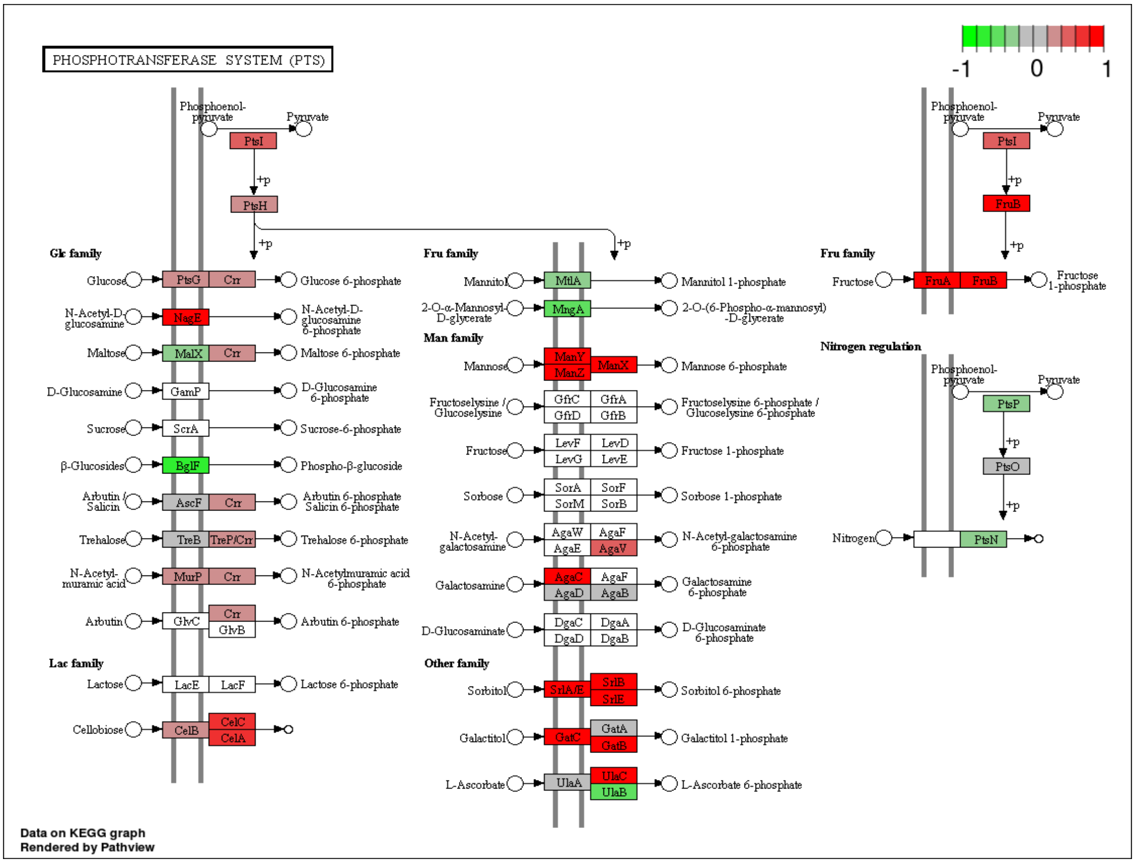

B Nonribosomal peptide biosynthesis

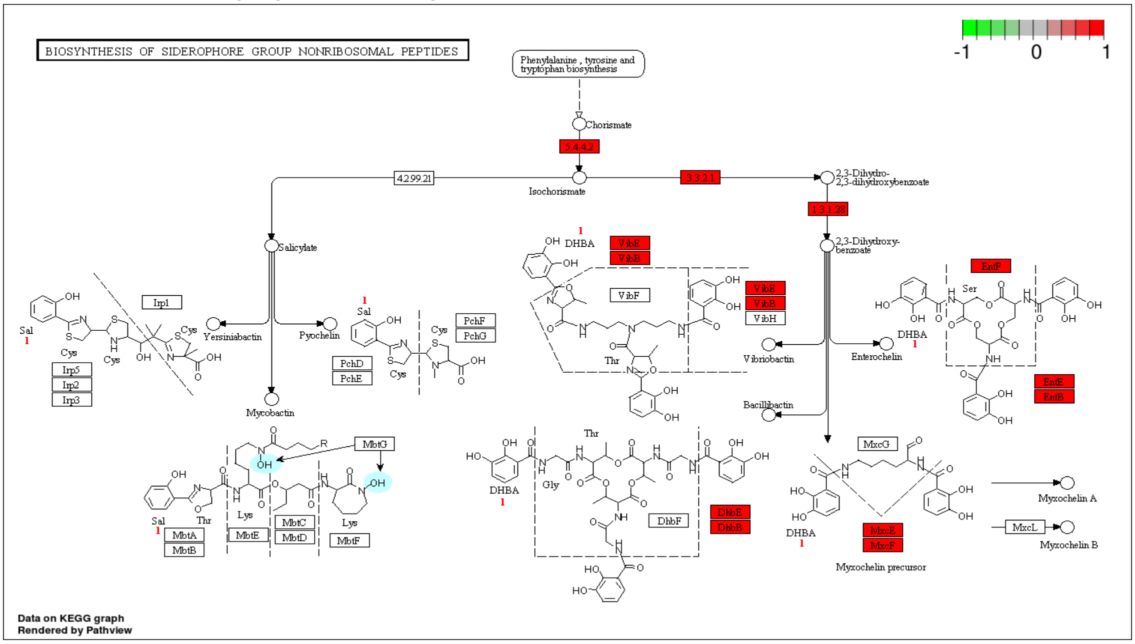

Fig. S8. KEGG pathway mapping of Cephalothin treated cells as determined by mRNA abundance analysis (P_{adj} value ≤0.05). (A) Significantly up regulated anerobic pathways and associated genes of phosphotransferase system and **(B)** non-ribosomal peptide biosynthesis. Colors that range from green to red are designated to genes extracted from log2 fold change of DEGs between untreated cells and Cephalothin treated cells.

Fig S9 Anaerobic pathways (up)

A Nitrogen metabolism

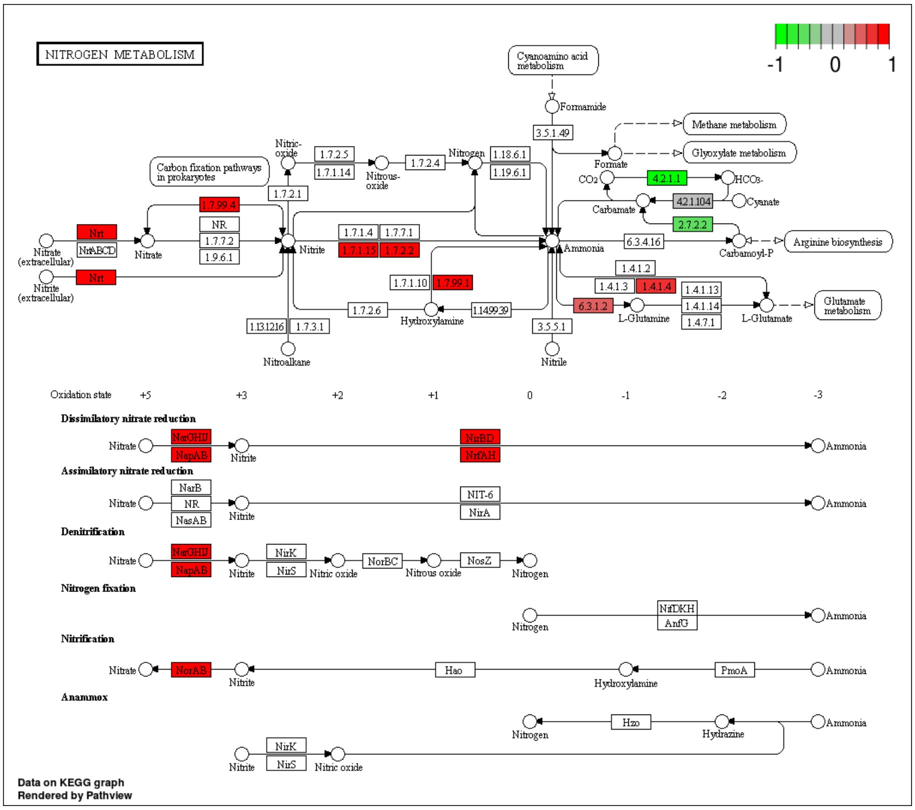

B Flagellar assembly

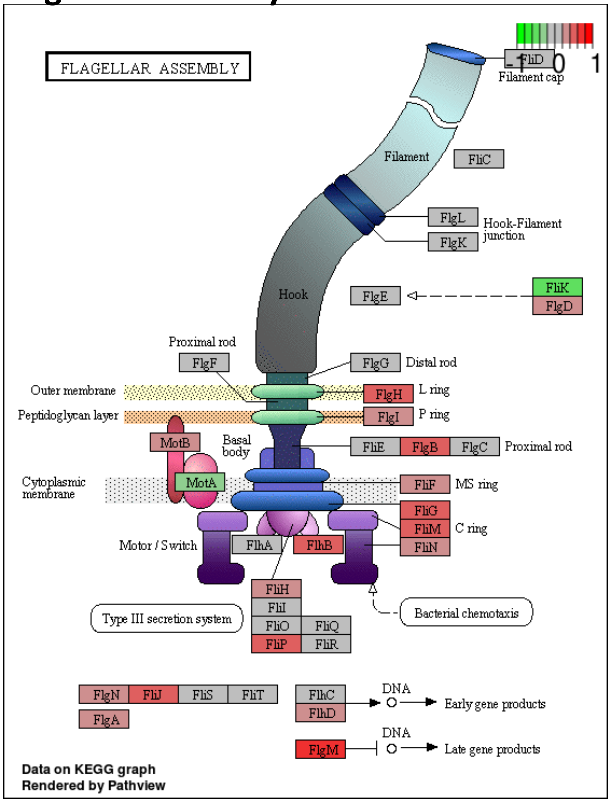

Fig. S9. KEGG pathway mapping of Cephalothin treated cells as determined by mRNA abundance analysis (P_{adj} value ≤0.05). (A) Significantly up regulated anerobic pathways and associated genes of nitrogen metabolism and **(B)** flagellar assembly. Colors that range from green to red are designated to genes extracted from log2 fold change of DEGs between untreated cells and Cephalothin treated cells.

Fig S10

Anaerobic pathways (up)

Bacterial chemotaxis

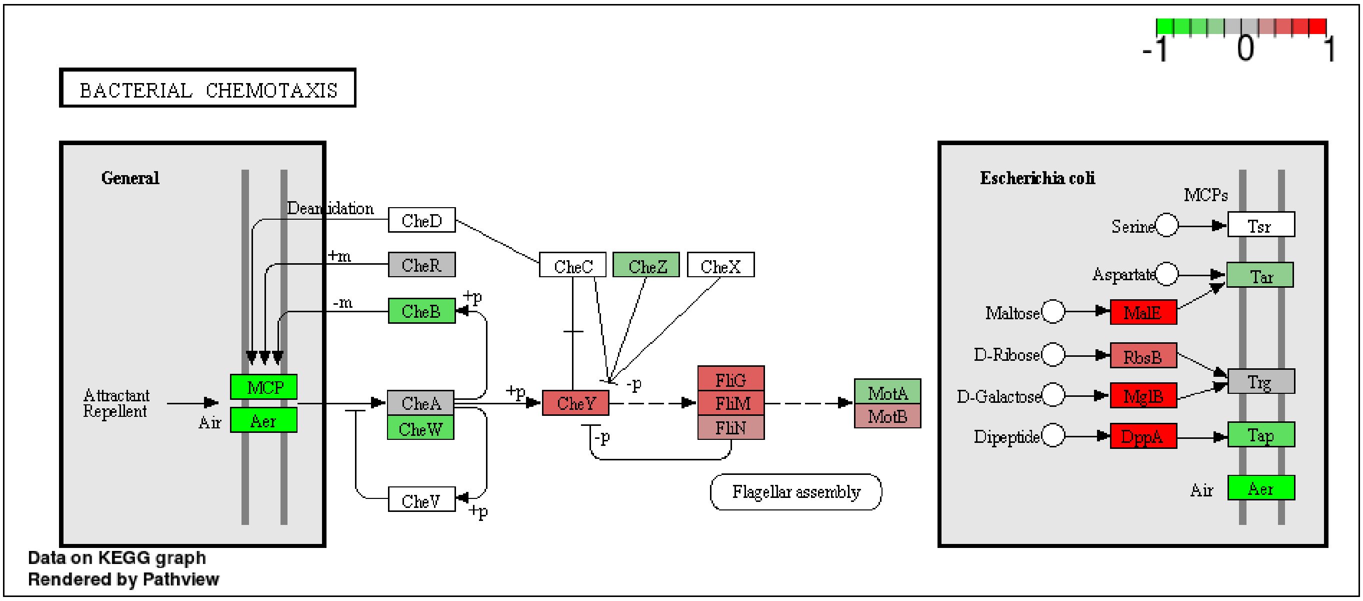

Fig. S10. KEGG pathway mapping of Cephalothin treated cells as determined by mRNA abundance analysis (P_{adj} value ≤0.05). (A) Significantly up regulated anaerobic pathways and associated genes of bacterial chemotaxis. Colors that range from green to red are designated to genes extracted from log2 fold change of DEGs between untreated cells and Cephalothin treated cells.

Fig S11

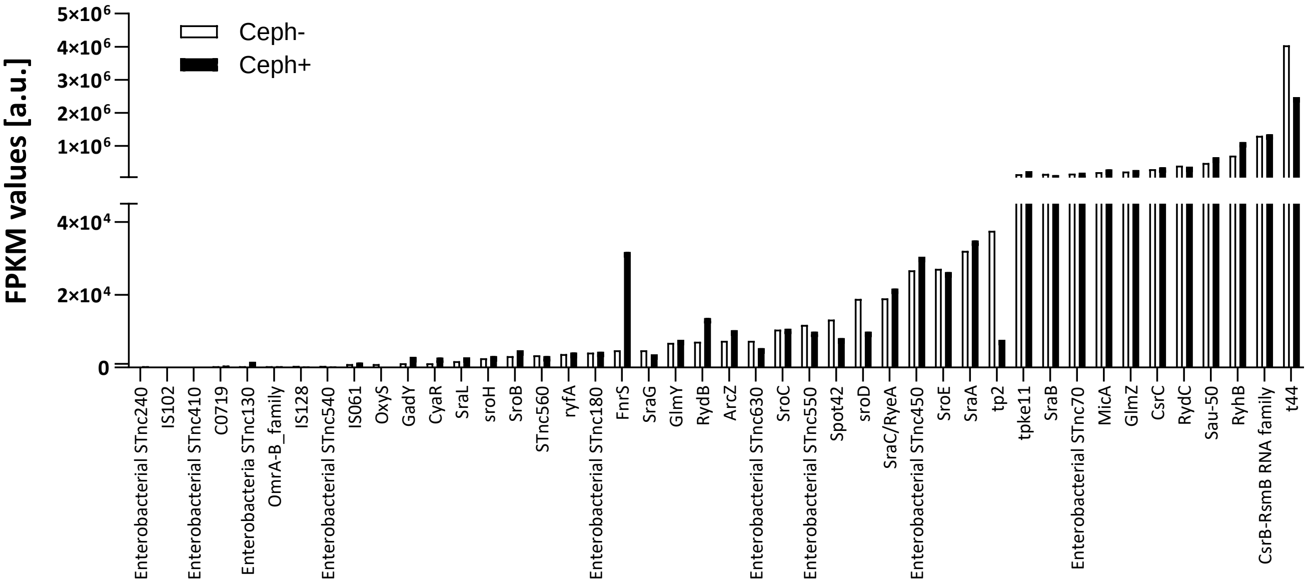

Fig. S11. Noncoding transcriptome analysis of untreated cells and Cephalothin treated cells. A total of 80 noncoding transcripts including sRNAs, tRNAs, antisense sRNAs and antitoxin RNAs were mapped, and sRNAs were plotted according to their FPKM values. All values are presented in Supplementary Table 3.

Fig S12

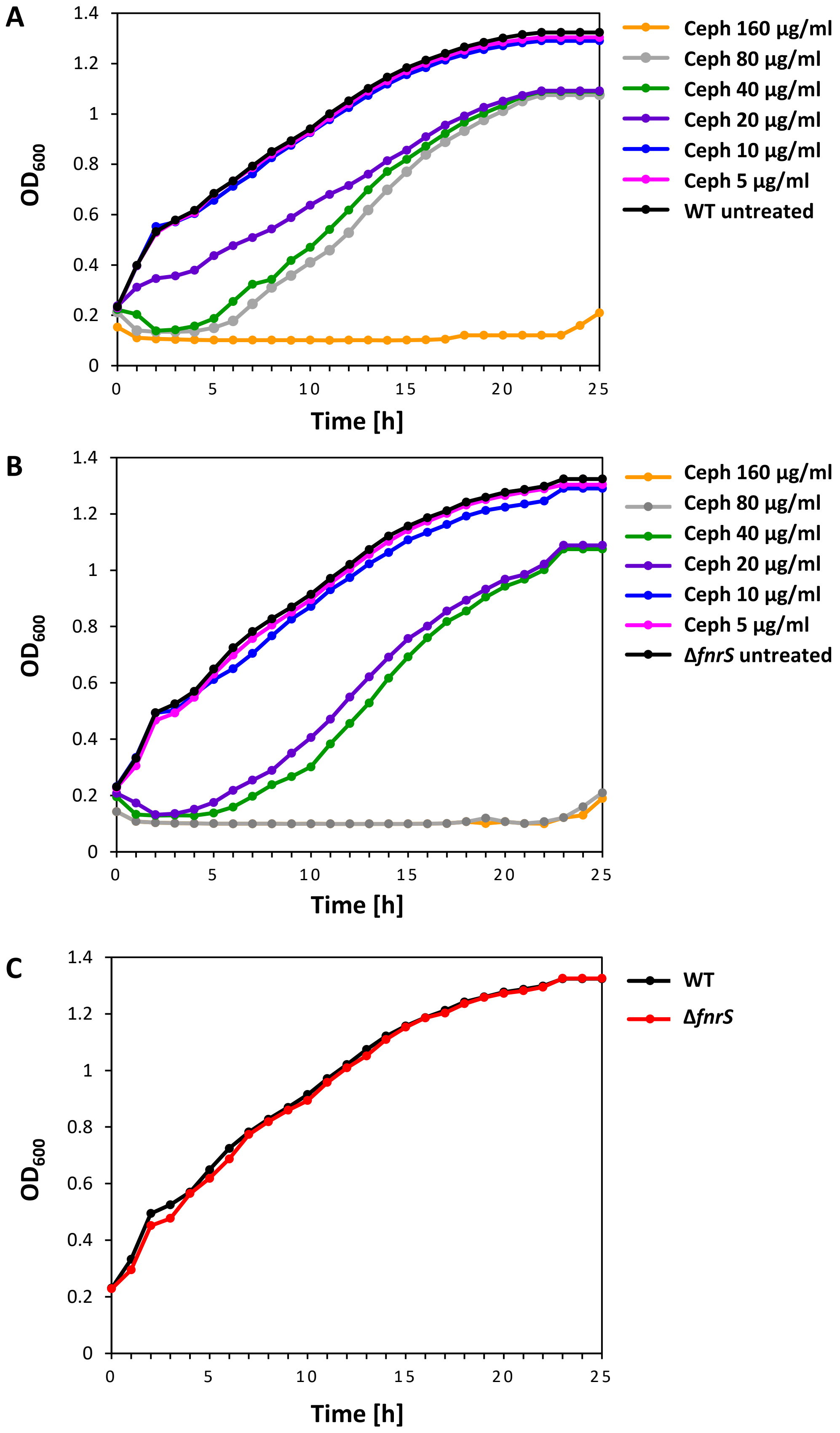

Fig. S12. Antibiotic-induced growth inhibition assay using wild-type (WT) and Δ*fnrS* mutant cells. Overnight grown cultures were freshly diluted at OD_{600} value of 0.05 and grown until OD_{600} value reached 0.3. **(A)** WT and **(B)** *ΔfnrS* mutant were treated with 160, 80, 40, 20, 10, and 5 µg/ml of Cephalothin. Untreated strain was used as control. Respective cultures (200 µl) were added to 96-well plate and measured over 24 h time course using Tecan Infinite 200 Pro multimode reader at 37°C. **(C)** Growth curve of WT and *ΔfnrS* mutant in antibiotic untreated conditions.

Fig S13

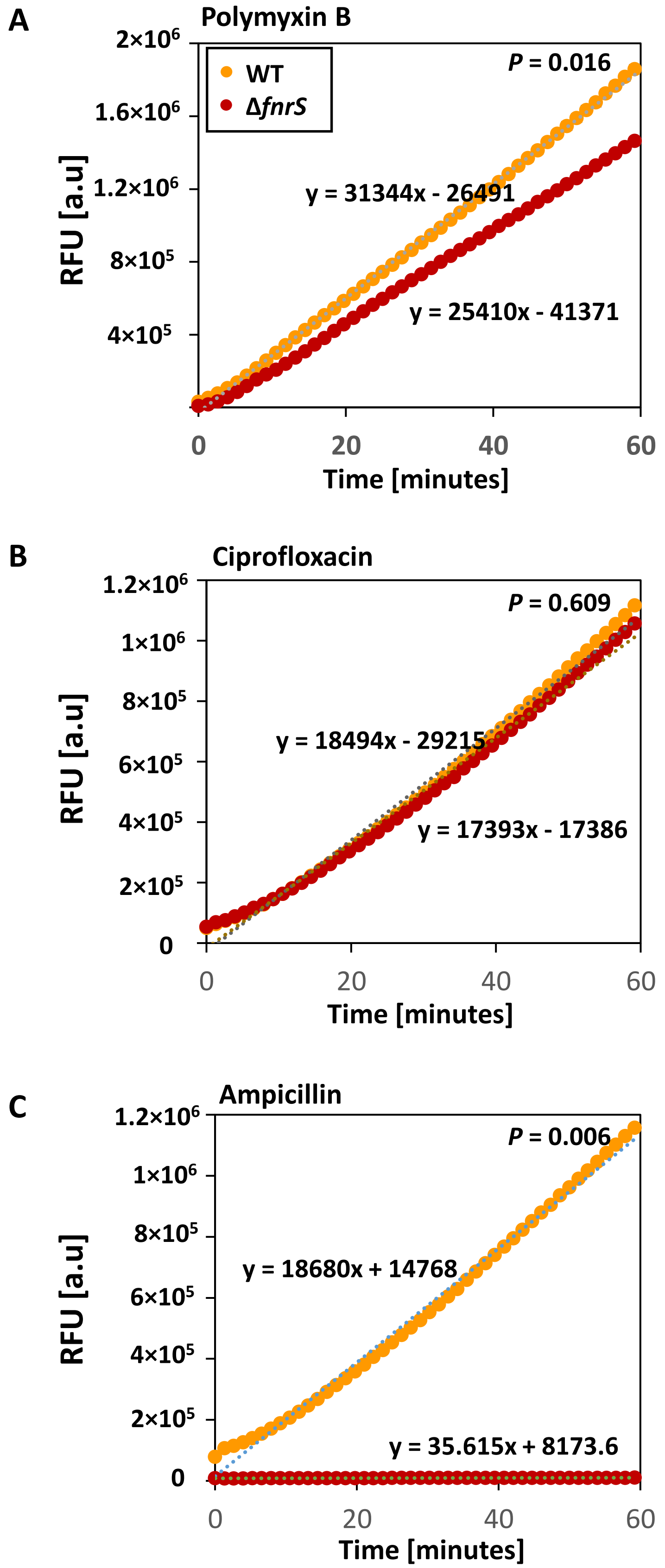

Fig. S13. (A-C) Fluorescence-based ROS detection was observed in wild-type and the Δ*fnrS* mutant. Bacterial cells were grown to OD_{600} value of 0.4 and treated with 0.2 µg/ml polymyxin B **(A)**, 10 ng/ml ciprofloxacin **(B)**, and 5 ng/ml ampicillin **(C)**. Then, 1 mM of ROS fluorescein dye H2DCFDA was immediately added. Relative fluorescence units (RFU) were determined using a fluorimeter. As ROS increased over 60 minutes, trend lines were plotted on the graph. The slopes of these trend lines were compared to determine differences. The experiment was performed in triplicate, and a representative figure is shown. *P*-values were determined with two-tailed Student's *t*-test.

Fig S14

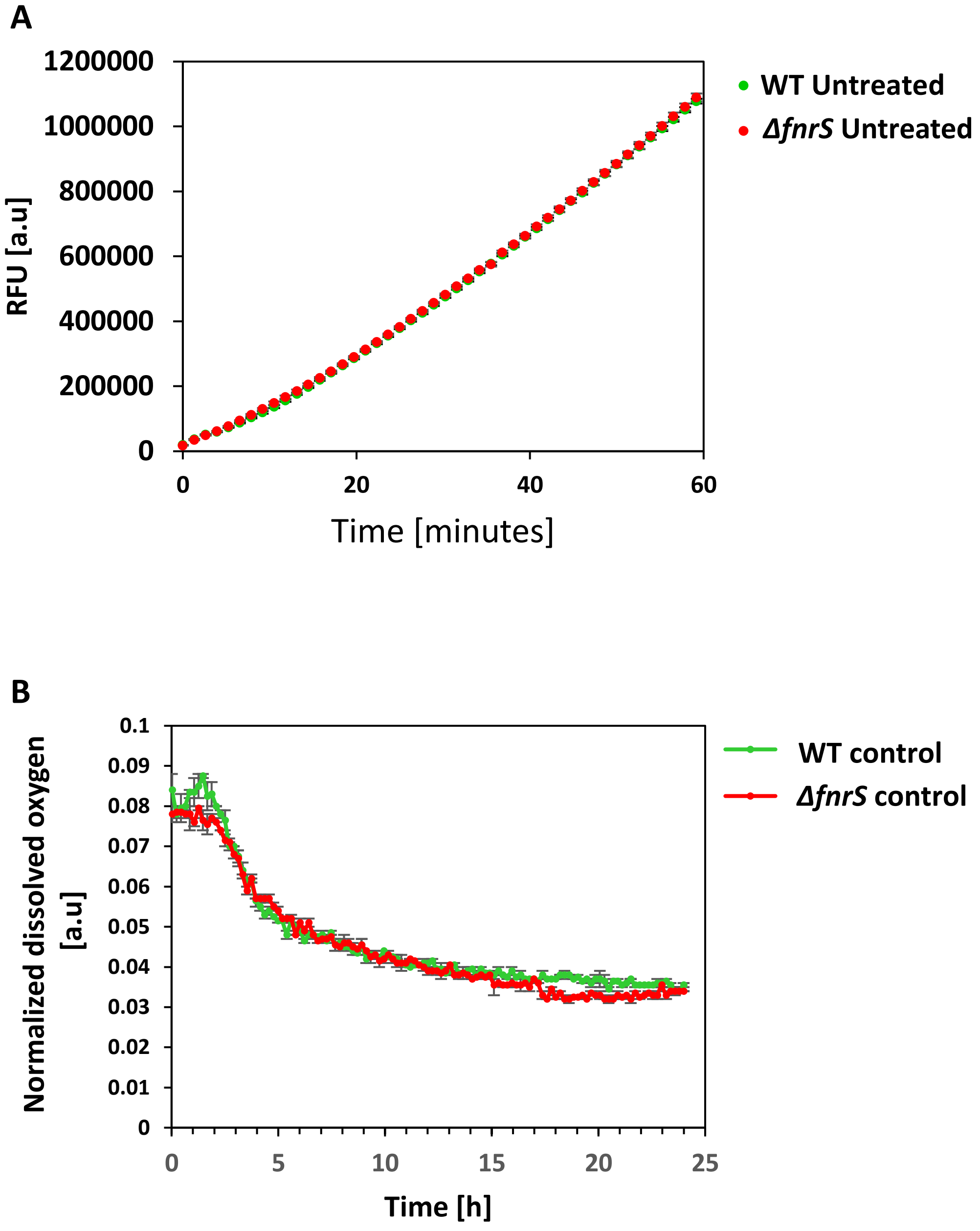

Fig. S14. Basal level ROS measurement and DOT measurement of wild type and Δ*fnrS* mutant. **(A)** Overnight grown cultures of wild type and *ΔfnrS* mutant were diluted to OD_{600} value of 0.05 and were allowed grow in 25 ml of LB media until OD600 value of 0.5. Cells were resuspended in PBS and were added with 1 µM of H2DCFDA. The total RFU were measured for 60 minutes and plotted as RFU over time in minutes. **(B)** Overnight grown cultures of wild type and *ΔfnrS* mutant were diluted to OD_{600} value of 0.05 and were grown in M9 minimal media supplemented with 0.2% casamino acids and 10 mM glucose until the OD600 value reached 0.3. DOT was measured using MTP-48-BOH flower plate on a Biolector I microbioreactor platform. Basal levels of ROS and DOT showed no observable difference between wild type and *ΔfnrS* mutant. Normalized dissolved oxygen levels were obtained by dividing the oxygen levels in culture by the biomass value.

Fig S15

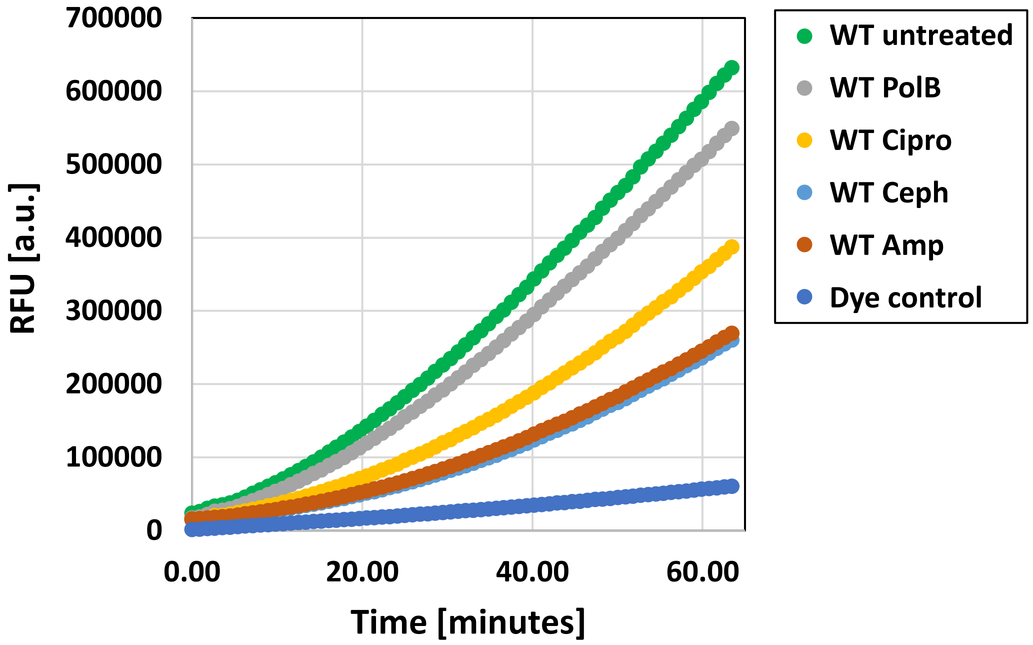

Fig. S12. ROS measurement wild type treated with antibiotics. Overnight grown cultures of wild type were diluted to OD_{600} value of 0.05 and were allowed grow in 25 ml of LB media until OD_{600} value of 0.5. Cells were resuspended in PBS and were added with 1 µM of H2DCFDA. Antibiotics were treated with 0.2 µg/ml polymyxin B (PolB), 10 ng/ml ciprofloxacin (Cipro), 20 µg/ml Celphalothin (Ceph), and 5 ng/ml ampicillin. The total RFU were measured for 60 minutes and plotted as RFU over time in minutes.

Supplementary Table S1

Table S1. The list DEGs of Cephalothin treated cells (P_{adj} value ≤0.05) showing the respective log2 fold change, *p*-value and *q*-value. A total of 41 genes were significantly upregulated and 8 genes were significantly downregulated.

Gene Name	Description	log2fc	p-value	q-value
tdcB	catabolic threonine dehydratase, PLP-dependent	6.46	5.00E-05	0.0069125
nirC	nitrite transporter	5.9	5.00E-05	0.0069125
nirB	nitrite reductase, large subunit, NAD(P)H-binding	5.46	5.00E-05	0.0069125
dcuC	anaerobic C4-dicarboxylate transport	5.25	5.00E-05	0.0069125
dmsB	dimethyl sulfoxide reductase, anaerobic, subunit B	5.02	5.00E-05	0.0069125
ansB	periplasmic L-asparaginase II	4.91	5.00E-05	0.0069125
narK	nitrate/nitrite transporter	4.84	5.00E-05	0.0069125
dppC	dipeptide/heme transporter	4.51	5.00E-05	0.0069125
dmsC	dimethyl sulfoxide reductase, anaerobic, subunit C	4.43	5.00E-05	0.0069125
tdcC	L-threonine/L-serine transporter	4.43	5.00E-05	0.0069125
nrfD	formate-dependent nitrite reductase, membrane subunit	4.43	0.00015	0.0187306
dmsA	dimethyl sulfoxide reductase, anaerobic, subunit A	4.32	5.00E-05	0.0069125
narG	nitrate reductase 1, alpha subunit	4.19	0.0003	0.027007
garD	(D)-galactarate dehydrogenase	4.18	5.00E-05	0.0069125
dppB	dipeptide/heme transporter	3.96	5.00E-05	0.0069125
tdcA	DNA-binding transcriptional activator	3.77	5.00E-05	0.0069125
yhbV	putative protease	3.69	5.00E-05	0.0069125
yjjI	hypothetical protein	3.68	0.0003	0.027007
gutM	DNA-binding transcriptional activator of glucitol operon	3.66	5.00E-05	0.0069125
ydeM	hypothetical protein	3.39	5.00E-05	0.0069125
napA	nitrate reductase, periplasmic, large subunit	3.35	5.00E-05	0.0069125
nrfA	nitrite reductase, formate-dependent, cytochrome	3.34	5.00E-05	0.0069125
nanC	N-acetylnuraminic acid outer membrane channel protein	3.28	5.00E-05	0.0069125
bssR	repressor of biofilm formation by indole transport regulation	3.15	5.00E-05	0.0069125
cadB	putative lysine/cadaverine transporter	3.13	5.00E-05	0.0069125
garL	alpha-dehydro-beta-deoxy-D-glucarate aldolase	3.06	5.00E-05	0.0069125
entH	thioesterase required for efficient enterobactin production	2.99	0.00055	0.042581
hcr	HCP oxidoreductase, NADH-dependent	2.88	0.00025	0.0254671
garK	glycerate kinase I	2.86	0.00015	0.0187306
garP	putative (D)-galactarate transporter	2.83	0.0002	0.02212
yfcC	putative inner membrane protein	2.81	0.0002	0.02212
yhjA	putative cytochrome C peroxidase	2.81	0.00025	0.0254671
dsdA	D-serine dehydratase	2.77	0.0004	0.0336609
nanM	N-acetylneuraminic acid mutarotase	2.72	0.0002	0.02212
yqeC	hypothetical protein	2.72	0.0003	0.027007
yeaH	UPF0229 family protein	2.7	0.0002	0.02212
entF	enterobactin synthase multienzyme complex component, ATP-dependent	2.59	0.0003	0.027007
yqeB	conserved protein with NAD(P)-binding Rossman fold	2.58	0.0006	0.0446654
yecH	DUF2492 family protein, function unknown	2.52	0.0005	0.0395
ysaA	putative hydrogenase, 4Fe-4S ferredoxin-type component	2.48	0.00045	0.0362906
ybdK	weak gamma-glutamyl:cysteine ligase	2.35	0.0006	0.0446654
mqo	malate dehydrogenase, FAD/NAD(P)-binding domain	-3.38	5.00E-05	0.0069125
putA	fused DNA-binding transcriptional regulator/proline dehydrogenase/pyrroline-5-carboxylate dehydrogenase	-3.37	5.00E-05	0.0069125
tdcF	putative reactive intermediate deaminase	-2.96	0.0001	0.0133483
ndk	multifunctional nucleoside diphosphate kinase and apyrimidinic endonuclease and 3'-phosphodiesterase	-2.72	0.00025	0.0254671
yncD	putative iron outer membrane transporter	-2.7	0.0003	0.027007
ydcI	putative DNA-binding transcriptional regulator	-2.68	0.00035	0.0301078
yigI	conserved protein, 4HBT family of thioesterases	-2.49	0.00035	0.0301078
rsxG	electron transport complex protein required for the reduction of SoxR	-2.52	0.00045	0.0362906

Supplementary Table S2

Table S2. Frequency distribution of GO terms. Revigo GO term analysis was performed for up- and down-regulated GO processes and the values obtained from semantic similarity measures were tabulated into percentage frequency and log10 *p*-value.

Description	Frequency	$\log_{10}$ *p*-value
**Upregulated processes**		
single-organism cellular process	43.24%	-1.222
localization	23.60%	-2.3455
oxoacid metabolic process	15.23%	-1.309
oxidation-reduction process	14.92%	-5.2211
single-organism transport	13.58%	-3.0311
cellular catabolic process	9.59%	-1.4312
generation of precursor metabolites and energy	4.08%	-4.3045
response to oxidative stress	2.46%	-1.1031
anaerobic respiration	1.25%	-8.4401
iron ion homeostasis	1.13%	-1.8657
reactive nitrogen species metabolic process	0.70%	-3.7905
nitrate assimilation	0.61%	-4.0334
response to hydrogen peroxide	0.52%	-2.3002
iron ion transport	0.49%	-1.6037
oligosaccharide transport	0.38%	-2.1462
nonribosomal peptide biosynthetic process	0.32%	-5.9666
anaerobic electron transport chain	0.29%	-2.2723
enterobactin metabolic process	0.26%	-8.4724
**Downregulated processes**		
cellular process	76.17%	-1.6601
cellular metabolic process	62.67%	-2.6882
primary metabolic process	55.78%	-1.7803
single-organism metabolic process	40.26%	-3.0241
cellular biosynthetic process	34.69%	-1.055
cellular nitrogen compound biosynthetic process	22.91%	-2.3988
oxidation-reduction process	14.92%	-4.7258
cation transport	5.50%	-3.399
ribose phosphate metabolic process	3.45%	-7.0605
purine-containing compound metabolic process	3.39%	-4.5513
ribonucleoside metabolic process	3.27%	-8.4225
pyrimidine-containing compound metabolic process	1.83%	-1.5494
aerobic respiration	1.36%	-4.6234
pyruvate metabolic process	1.19%	-1.6371
hydrogen ion transmembrane transport	0.84%	-7.1314

Supplementary Table S3

Table S3. Noncoding transcriptome analysis of untreated cells and Cephalothin treated cells. A total of 80 noncoding transcripts including sRNAs, tRNAs, antisense sRNAs and antitoxin RNAs were mapped, and sRNAs were plotted according to their FPKM values.

sRNAs	Cephalothin Untreated (FPKM values)	Cephalothin Treated (FPKM values)
t44	4041840	2473390
CsrB-RsmB RNA family	1297790	1338590
RyhB	702229	1110970
Sau-50	481059	648340
RydC	396246	360462
CsrC	287590	340560
GlmZ	219912	263373
MicA	202666	284816
Enterobacterial STnc70	154260	184868
SraB	146664	109945
tpke11	142471	227042
**tp2**	**37555**	**7605**
SraA	32047	34945
SroE	27097	26262
Enterobacterial STnc450	26744	30467
SraC/RyeA	19039	21667
sroD	18851	9856
Spot42	13136	8074
Enterobacterial STnc550	11717	9892
SroC	10400	10661
Enterobacterial STnc630	7331	5275
ArcZ	7320	10223
RydB	7107	13603
GlmY	6790	7557
SraG	4767	3645
**FnrS**	**4746**	**31757**
Enterobacterial STnc180	4070	4346
ryfA	3649	4087
STnc560	3373	3131
SroB	3131	4731
sroH	2557	3086
SraL	1724	2766
CyaR	1165	2747
GadY	1162	2863
OxyS	948	0
IS061	915	1333
Enterobacterial STnc540	426	250
IS128	414	297
OmrA-B_family	390	366
Enterobacteria STnc130	362	1604
C0719	361	525
Enterobacterial STnc410	77	142
IS102	34	94
Enterobacterial STnc240	0	390

Supplementary Table S4

Table S4. Strains, plasmids and primers used in this study

Name	Descriptions	Source/reference
**Strains**		
MG1655	MG1655 wild type	Laboratory stock
GSO402	MG1655 DE(*fnrS::kan*)	Durand and Storz, 2010
BW25113	*rrnB3* DE*lacz4787 hsdR514* DE(*araBAD*)*567* DE(rhaBAD)568 *rph-1*	Datensko and Wanner, 2000
BW25113*Δfnr*	DE*(fnr::kan) rrnB3* DE*lacz4787 hsdR514* DE(*araBAD*)*567* DE(rhaBAD)568 *rph-1*	Datensko and Wanner, 2000
BW25113*ΔarcA*	DE*(arcA::kan) rrnB3* DE*lacz4787 hsdR514* DE(*araBAD*)*567* DE(rhaBAD)568 *rph-1*	Datensko and Wanner, 2000
**Plasmids**		
pBR-plac	Placpromoter based expression vector, amp^R	Guillier et. al., 2006
pBRlac-FnrS	AatII-EcoRI FnrS containing fregment cloned into pBR-plac	Guillier et. al., 2006
pEM1396	pBAD-*ryhB*	Massé et. al., 2003

Primers

Gene name	Forward (5'–3')	Reverse (5'–3')	Efficiency(%)	R^2
rrsA (16S rRNA)	GCTACAATGGCGCATACAAA	TTCAT GGAGT CGAGT TGCAG	94.95	0.990
tdcA	GCGCAACTGTCTTCGTTCTT	TCGGGACTTACTGGCTACCA	99.49	0.992
nirB	CATCCAGAAAGACGGCACCT	TTTCTGTGCGCCAAACATCG	98.27	0.999
dmsA	GCGATGACAATTACGACGGC	GCTCGAATTTGCCTTCACCG	96.05	0.999
narK	GGTCTGAATGGTGGTCTGGG	AAGGAACGGCACCCATATCC	104.00	0.993
ddpB	GATTATCGTCCATGCGCTGC	CAGTGCGTCAATCAACCAGC	95.93	0.999
garD	ATCCATCTGCTGACACCACG	CAGACCGCCTTTCTTGTTGC	96.42	0.999
nrfA	GTTAACCCTTTCCCGTCCGT	ACTGACCGCAAACCATCGAT	98.76	0.999
nanC	TTCACCTTCGCATGGCAAAC	TGCCGTTGTAAACACCCTGA	93.31	0.999
dcuC	TTGCCGCTTACATGACCCAT	CCAGAGACATCAGACAGGCG	95.01	0.999
ansB	ACAGTGGGTAAAGTTGGCGT	ATCGTTCATGTCCTGGGAGC	99.46	0.999
FnrS	ATGCAACGTCAAGCGATG	AGCCGACTCATCAAAGTC	98.84	0.999
Tp2	TGAGCGTACCTGAAGTGG	GCTGGATGGTCGTTTCTAC	96.79	0.999
